# Optimization of *Pichia pastoris* Expression System for High-Level Production of Margatoxin

**DOI:** 10.3389/fphar.2021.733610

**Published:** 2021-09-29

**Authors:** Muhammad Umair Naseem, Gabor Tajti, Attila Gaspar, Tibor G. Szanto, Jesús Borrego, Gyorgy Panyi

**Affiliations:** ^1^ Department of Biophysics and Cell Biology, Faculty of Medicine, University of Debrecen, Debrecen, Hungary; ^2^ Department of Inorganic and Analytical Chemistry, Faculty of Science and Technology, Institute of Chemistry, University of Debrecen, Debrecen, Hungary

**Keywords:** *Pichia pastoris*, patch-clamp, margatoxin, recombinant expression, Kv1.3 blocker, CD4^+^ T_EM_ cells

## Abstract

Margatoxin (MgTx) is a high-affinity blocker of voltage-gated potassium (Kv) channels. It inhibits Kv1.1–Kv1.3 ion channels in picomolar concentrations. This toxin is widely used to study physiological function of Kv ion channels in various cell types, including immune cells. Isolation of native MgTx in large quantities from scorpion venom is not affordable. Chemical synthesis and recombinant production in *Escherichia coli* need *in vitro* oxidative refolding for proper disulfide bond formation, resulting in a very low yield of peptide production. The *Pichia pastoris* expression system offers an economical approach to overcome all these limitations and gives a higher yield of correctly refolded recombinant peptides. In this study, improved heterologous expression of recombinant MgTx (rMgTx) in *P. pastoris* was obtained by using preferential codons, selecting the hyper-resistant clone against Zeocin, and optimizing the culturing conditions. About 36 ± 4 mg/L of >98% pure His-tagged rMgTx (TrMgTx) was produced, which is a threefold higher yield than has been previously reported. Proteolytic digestion of TrMgTx with factor Xa generated untagged rMgTx (UrMgTx). Both TrMgTx and UrMgTx blocked the Kv1.2 and Kv1.3 currents (patch-clamp) (*K*_*d*_ for Kv1.2 were 64 and 14 pM, and for Kv1.3, 86 and 50 pM, respectively) with comparable potency to the native MgTx. The analysis of the binding kinetics showed that TrMgTx had a lower association rate than UrMgTx for both Kv1.2 and Kv1.3. The dissociation rate of both the analogues was the same for Kv1.3. However, in the case of Kv1.2, TrMgTx showed a much higher dissociation rate with full recovery of the block than UrMgTx. Moreover, in a biological functional assay, both peptides significantly downregulated the expression of early activation markers IL2R and CD40L in activated CD4^+^ T_EM_ lymphocytes whose activation was Kv1.3 dependent. In conclusion, the authors report that the *Pichia* expression system is a powerful method to produce disulfide-rich peptides, the overexpression of which could be enhanced noticeably through optimization strategies, making it more cost-effective. Since the presence of the His-tag on rMgTx only mildly altered the block equilibrium and binding kinetics, recombinant toxins could be used in ion channel research without removing the tag and could thus reduce the cost and time demand for toxin production.

## Introduction

Voltage-gated potassium (Kv) channels are present in a variety of cells and tissues where they regulate multiple physiological processes, including cardiac function, neural excitability, muscle contraction, cell proliferation, cell volume control, and hormonal secretion (Coetzee et al., 1999; Giangiacomo et al., 2004). In recent years, it has been shown that modulating the function of Kv channels may have therapeutic potential in cardiac arrhythmia, diabetes, asthma, inflammation, neuronal disorders, and T-cell–mediated autoimmune diseases and anti-tumor immunity (Chandy et al., 2004; Panyi et al., 2006; Jiménez-Vargas et al., 2012; Panyi et al., 2014; Kazama, 2015; Rubaiy, 2016; Yang and Nerbonne, 2016; Hofschröer et al., 2021). Kv1.3 plays a key role in pathogenesis of autoimmune diseases, e.g., multiple sclerosis, rheumatoid arthritis, and type-1 diabetes by triggering the activation and proliferation of T effector memory (T_EM_) cells (Chandy et al., 2004; Beeton et al., 2011a; Lam and Wulff, 2011; Chi et al., 2012; Toldi et al., 2013; Koshy et al., 2014). Since selective block of Kv1.3 suppresses the proliferation of T_EM_ cells, Kv1.3 has become an attractive immunomodulatory drug target in treating autoimmune diseases. Numerous peptide toxins have been derived over the past few decades from scorpion venom, which target and modulate Kv channel functions. These peptides consist of 20–80 residues and 3–4 conserved disulfide bridges to stabilize their tertiary structures that are responsible for specific interaction with ion channels (Shen et al., 2017; Tajti et al., 2020). Of these, the scorpion toxins from the α-KTx family, e.g., charybdotoxin (α-KTx 1.1), margatoxin (α-KTx 2.2), and Vm24 (α-KTx 23.1), have provided the platform for studying the pharmacological, physiological, and structural characteristics of different subtypes of K^+^ channels (Tenenholz et al., 2000; de la Vega et al., 2003; Han et al., 2011; Veytia-Bucheli et al., 2018). Although scorpion toxins inhibit Kv1.3 at nanomolar concentrations, they often show off-target effects by blocking other Kv1.x ion channels (Kalman et al., 1998; Bagdány et al., 2005). Therefore, for therapeutic purposes, peptide engineering is needed to design a peptide toxin with a higher selectivity and potency for Kv1.3. ShK-186 (Dalazatide), one of the engineered analogs of ShK toxin (isolated from sea anemone), is a potent and selective inhibitor of Kv1.3 that is under clinical trials for the treatment of multiple autoimmune disorders (Pennington et al., 2015; Tarcha et al., 2017; Tajti et al., 2020; Wang et al., 2020).

For structure–function relationship studies of engineered peptides and therapeutic applications, a large amount of peptide toxins is needed. Natural crude venom yields a limited amount of peptide toxins that are usually inadequate for biological analysis, while the chemical synthesis offers an expensive approach to produce disulfide-rich peptides and their analogs (Jensen et al., 2009). On the other hand, the heterologous protein expression system is a cost-effective and the most widely used technique to produce large quantities of recombinant proteins. Still, intracellular recombinant expression of eukaryotic origin proteins in bacteria has limitations, such as insoluble expression, a lack of posttranslational modifications, and disulfide bond formation (Rudolph and Lilie, 1996). However, the periplasmic expression or use of engineered *Escherichia coli* strains, which are capable of disulfide bond formation, usually produce refolded soluble proteins, but their yield is very low (Lobstein et al., 2012; Klint et al., 2013). To overcome all these limitations, the *Pichia pastori*s expression system was introduced that provides an efficient and economical approach, and produces heterologous proteins in correctly refolded form with disulfide bridges (White et al., 1994; Anangi et al., 2012). High-level growth in a simple medium, ease of genetic manipulation, and capability of performing posttranslational modifications are the other advantages of this system. Furthermore, the recombinant proteins are secreted directly into the medium with very few endogenous proteins, which simplifies the downstream processing (Macauley-Patrick et al., 2005; Cregg, 2007). The overexpression of heterologous proteins in this system can be enhanced considerably by codon optimization, screening for multiple copy integrant, choosing an efficient promoter, and optimizing fermentation conditions, such as biomass production, pH of the medium, induction duration, and percentage of methanol induction (Macauley-Patrick et al., 2005; Yu et al., 2010; Yu et al., 2013). In comparison to the yeast expression system, production of recombinant proteins in insect cell and animal cell cultures is complicated and expensive (Escoubas et al., 2003).

Margatoxin (MgTx) is a 39-amino-acid peptide toxin isolated from the venom of the scorpion *Centruroides margaritatus*. Its 3D structure consists of an α-helix and three antiparallel β-strands stabilized by three disulfide bonds. This peptide toxin has been widely used for structural and functional characterization of Kv1.x ion channels in various cell types and tissues, as it inhibits Kv1.2 and Kv1.3 ion channels with high potency in picomolar concentrations (Garcia-Calvo et al., 1993; Bartok et al., 2014). Previous studies report the production of recombinant MgTx (rMgTx) in *E. coli* and *P. pastoris* with a yield of 3–4 mg (Garcia-Calvo et al., 1993; Johnson et al., 1994) and 12–15 mg per liter (Anangi et al., 2012), respectively.

In this work, the *P. pastoris* expression system was optimized to achieve a high-level expression of rMgTx. First, biased codon optimization was used to select the clone showing hyper-resistance against the selection marker. The fermentation conditions (pH of the medium, induction time course, and methanol induction) were then optimized to get a high yield (36 mg/L) of the peptide. After purification, the N-terminal His-tag was removed by using factor Xa protease. It was found that both versions (tagged and untagged) of rMgTx inhibited the hKv1.3 and hKv1.2 channels in picomolar concentrations. Both peptides also downregulated IL2R and CD40L expression in activated CD4^+^ T_EM_ cells through Kv1.3 blockade. Moreover, in this study, the influence of the N-terminal His-tag (additional residues) of rMgTx on binding kinetics to hKv1.3 and hKv1.2 was studied.

## Materials and Methods

### Construction of Plasmid

**FIGURE 1:**
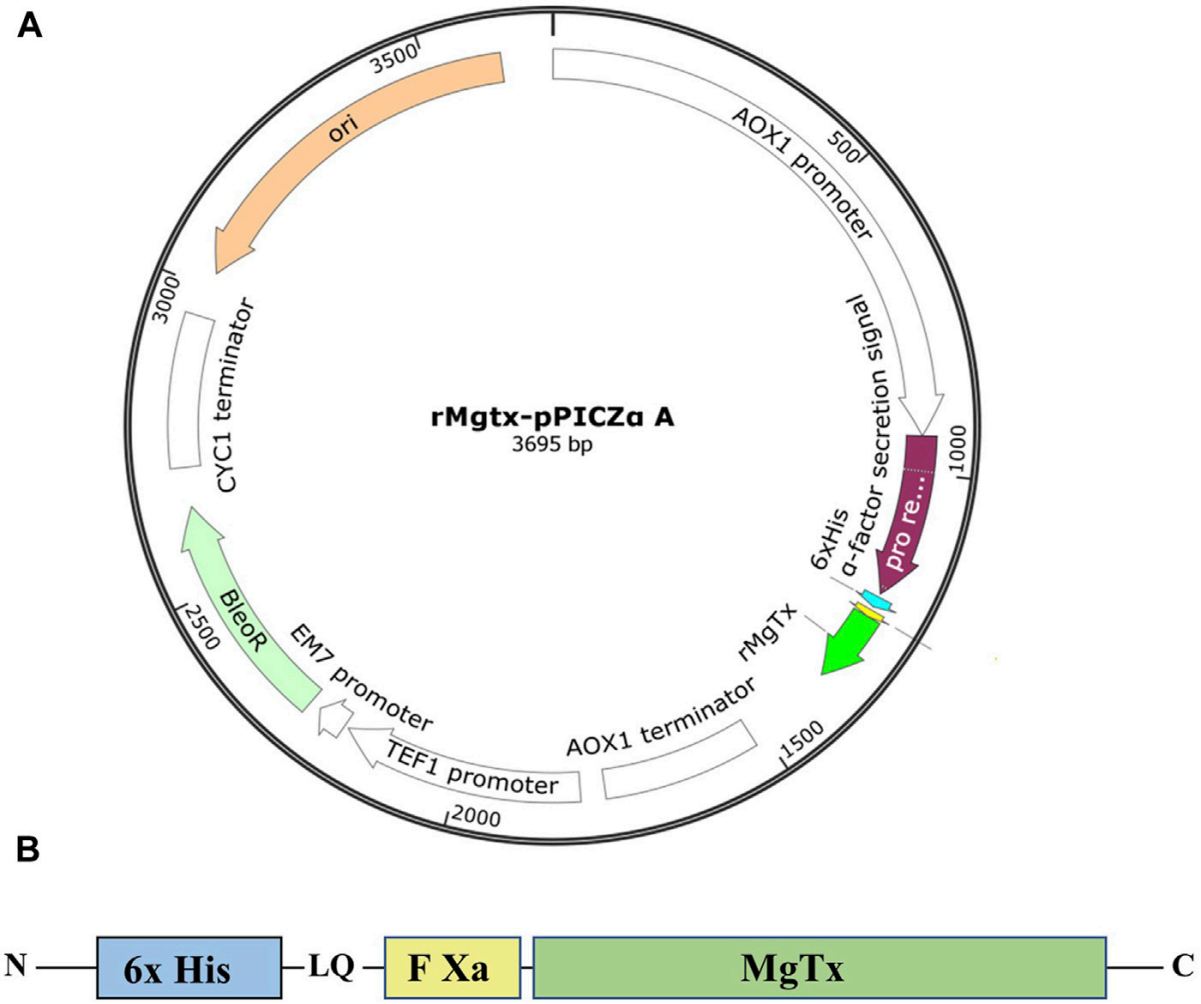
**(A)** Graphical representation of recombinant plasmid TrMgTx–pPICZαA designed using the SnapGene^®^ tool. **(B)** Schematic demonstration of the TrMgTx cassette.

The amino acid sequence of MgTx was retrieved from the online protein (Uniprot P40755) database. The MgTx gene cassette was designed by placing the 6xHis-tag at the N-terminal to facilitate purification, and factor Xa protease site was introduced in between them to obtain native N-terminal MgTx, as demonstrated in Figure 1. The codon-optimized DNA sequence of this MgTx cassette for *P. pastoris* was generated according to the codon usage database available at www.kazusa.or.jp/codon and synthesized from Integrated DNA Technologies, Belgium. The codon-optimized MgTx cassette was cloned into yeast expression vector pPICZα A (Invitrogen, United States) by using *EcoRI* and *XbaI* restriction sites. In-frame ligation and nucleotide sequence of MgTx was confirmed by DNA sequencing by using plasmid-specific primers and aligning the obtained DNA sequence with the theoretical sequence of MgTx.

### Transformation of *Pichia pastoris* X-33 and Selection of Hyper-Resistant Transformants Against Zeocin

The expression plasmid was linearized by digesting with *SacI* endonuclease enzyme and transformed into *P. pastoris* X-33 competent cells using Pichia EasyComp Transformation Kit (Invitrogen, United States), following the protocol specified by the manufacturer. Transformed X-33 cells were spread on YPD agar plate (2% peptone, 1% yeast extract, 2% agar, 2% dextrose, and pH 7.0) containing 100 µg/ml of Zeocin. After 3-day incubation at 28°C, 24 prominent colonies were regrown on YPD plates supplemented with progressively increasing Zeocin 500, 1000, and 2000 µg/ml for the selection of the clone showing hyper-resistance against Zeocin. To confirm the integration of expression construct into the genome of *Pichia* transformants, survived on 2000 µg/ml Zeocin, colony PCR was performed by using plasmid-specific primers.

### Time Course Study of MgTx Expression and Optimization of pH of the Medium and Methanol Induction

A selected clone from the YPD plate containing 2000 µg/ml of Zeocin was grown overnight in 5 ml of the YPD medium and diluted the next day to an OD_600_ = 0.2 in 5 ml of BMGY medium (1% yeast extract, 2% peptone, 100 mM potassium phosphate, pH 6.0, 1.34% YNB, $4\times 10^{-5}$ % biotin, and 2% glycerol) for biomass production at 30°C with constant shaking (230 rpm) until the OD_600_ reached between 15 and 20 (after 24–36 h). Cells were collected by centrifugation, resuspended in 5 ml of BMMY induction medium (same as BMGY with 0.5% methanol instead of glycerol), and grown for 5 days at 28°C with constant shaking (230 rpm). Absolute methanol (MeOH, 0.5%) was added every 24 h to maintain the induction, except when MeOH concentration dependence of induction was studied. To find the suitable amount of methanol induction, cells were induced with 0.5, 1, and 1.5% MeOH, and for pH optimization, cells were grown in media of different pH, i.e., 5, 6, and 7, without buffering. About 15 µl of the supernatant samples were taken at indicated time points and analyzed on 16% tricine–SDS-PAGE. The amount of His-tagged rMgTx (TrMgTx) in the gel image was determined by comparing the band intensities with the standards (TrMgTx with known concentration) using Image Lab tool (Bio-Rad). All the experiments were run in triplicates.

### Large-Scale Fed-Batch Fermentation and Purification of TrMgTx

Large-scale flask-level production was executed following the optimized conditions as described earlier. The clone with the highest expression level of TrMgTx was inoculated in a 2-L flask containing 250 ml of the BMGY medium, and when the OD_600_ reached between 15 and 20, the cells were shifted to 250 ml of the BMMY induction medium and induced with 0.5% MeOH for 72 h.

Two-step purification was used to efficiently isolate secreted TrMgTx from the culture. The cultured supernatant was collected by high-speed centrifugation, 2× diluted with buffer (50 mM potassium phosphate, pH 7.4), and 60 mM imidazole was added. The filtered supernatant was loaded on pre-equilibrated His-trap column packed with Ni Sepharose^TM^ High Performance affinity media (GE Healthcare, United Kingdom) with a binding buffer (50 mM potassium phosphate, 300 mM NaCl, and pH 7.4) at a flow rate of 3 ml/min using the liquid chromatography (LC) system (Shimadzu, Germany). After washing the column with three column volumes (CVs) of wash buffer (binding buffer + 60 mM imidazole), proteins were eluted by running three CVs of the elution buffer (binding buffer + 500 mM imidazole) and an additional three CVs of 1 M imidazole in isocratic mode. Fractions collected from the affinity column were directly applied on reversed-phase C_18_ semi-prep column (10 mm) × 250 mm, 5 µM bead size, 300 Å pore size, Vydac® 218 TP, HiChrom, United Kingdom) using Prominence HPLC System (Shimadzu, Germany) at a flow rate of 1 ml/min. Then, a linear gradient of 10–30% of Solvent B (0.1% TFA in 95% acetonitrile) in Solvent A (0.1% TFA in deionized distilled water) was run for 30 min. Absorbance was monitored at 230 nm with a PDA detector. The peak fractions were collected manually and tested on 16% tricine–SDS-PAGE. The purity level was judged by reloading the fraction on the reversed-phase C_18_ analytical column and was calculated by the equation: (area under the peak of interest)/(cumulated area under all the peaks) × x 100.

### SDS-PAGE and Western Blot

16% Tricine–SDS-PAGE was performed as described hitherto (Schägger, 2006). The protein sample was mixed with tricine sample buffer (Bio-Rad) at 1:1, incubated at 95°C for 5 min, and subsequently centrifuged at 10,000 rpm for 30 s before loading. Electrophoresis was carried out at constant 120 V for 90 min. For protein visualization, the gel was stained with Coomassie Brilliant Blue G-250 for 45 min and then destained by using 40% methanol and 10% acetic acid mixture for 2–3 h.

For Western blotting, the resolved proteins were electrotransferred in wet conditions onto a charged Immobilon-P PVDF membrane (Merck, Germany). Non–specific binding of antibodies in the subsequent steps was prevented by membrane blocking with 5% (w/v) skim milk in TBST (50 mM Tris-HCl, pH 7.5, 150 mM NaCl, and 0.1% Tween 20), overnight at 4°C. The washed membrane was probed with mouse anti-histidine monoclonal antibodies conjugated with horseradish peroxidase (Bio-Rad, CA, United States) in TBST (1:2,500) and incubated for 1 h at room temperature. The bands were visualized using Pierce™ enhanced chemiluminescent (ECL) substrate (Thermo Scientific, MA, United States).

### Cleavage of His-Tag From TrMgTx

Hexahistidine residues fused at the N-terminal of TrMgTx were cleaved with factor Xa protease (Thermo Scientific, United States Cat# 32,521). About 300 µg of TrMgTx was mixed with factor Xa at an enzyme-to-substrate ratio of 1:100 in TBS buffer (50 mM Tris, 100 mM NaCl, 6 mM CaCl_2_, pH 8.0) and incubated overnight at 25°C. The next day, the samples treated with or without enzyme were analyzed on 16% tricine/6 M urea–SDS-PAGE. To purify untagged rMgTx (UrMgTx), the cleaved His-tag and undigested peptide fragments were captured by using pre-charged Ni^+^ beads, centrifuged at a high speed for 1 min to remove the beads, and the supernatant was loaded on a reversed-phase C_18_ analytical column (4.6 mm × 250 mm, 5 µM bead size, Vydac® 218TP) using the HPLC system (Shimadzu, Germany) and eluted with a linear gradient of 10–30% of Solvent B (0.1% TFA in 95% acetonitrile) in Solvent A (0.1% TFA in deionized distilled water) that was run for 25 min.

### Mass Spectrometry Analyses

Mass spectrometric determinations were performed with an ESI-QTOF-MS instrument (maXis II UHR ESI-QTOF MS, Bruker, Bremen, Germany). The mass spectrometer was operated in a positive ionization mode; 0.5 bar nebulizer pressure, 200°C dry gas temperature, 4 L/min dry gas flow rate, 4000 V capillary voltage, 500 V end plate offset, 1 Hz spectra rate, and 500–2,500 m/z mass range were applied. ESI tuning mix (Agilent) calibrant injected after each run enabled internal m/z calibration. Mass spectra were processed and evaluated by Compass Data Analysis version 4.4 (Bruker).

### Cells

The human venous blood from anonymized healthy donors was obtained from a blood bank. The peripheral blood mononuclear cells were isolated through Histopaque1077 (Sigma-Aldrich Hungary, Budapest, Hungary) density gradient centrifugation. Cells obtained were resuspended in RPMI 1640 medium containing 10% fetal calf serum (Sigma-Aldrich), 100 µg/ml penicillin, 100 µg/ml streptomycin, and 2 mM_l_ glutamine, seeded in a 24-well culture plate at a density of $5\;x\;10^{5}$ cells per ml, and grown in a 5% CO_2_ incubator at 37°C for 2–5 days. Phytohemagglutinin A (Sigma-Aldrich) was added in 5, 7, and 10 µg/ml concentrations to the medium to boost the potassium ion channel expression.

CHO cells were transiently transfected with the vector pCMV6-GFP (OriGene Technologies, Germany) encoding the human Kv1.2 ion channel using Lipofectamine 2000 (Invitrogen, Carlsbad, CA), following the manufacturer's protocol, and grown under the standard conditions as used previously (Bagdány et al., 2005). GFP-positive transfectants were identified with Nikon TE 2000U fluorescence microscope (Nikon, Tokyo, Japan), and currents were recorded after 24 h post transfection.

### Electrophysiology

Electrophysiological measurements were performed by using the patch-clamp technique in whole-cell or outside-out patch configuration and voltage clamp mode using the Multiclamp 700B amplifier and Axon Digidata1440 digitizer (Molecular Devices, Sunnyvale, CA). Micropipettes were pulled from GC150F-15 borosilicate capillaries (Harvard Apparatus Kent, United Kingdom), resulting in 3–5 MΩ resistance in the bath solution. The extracellular solution contained 145 mM NaCl, 5 mM KCl, 1 mM MgCl_2_, 2.5 mM CaCl_2_, 5.5 mM glucose, and 10 mM HEPES, with a pH of 7.35 and an osmolarity between 302 and 308 mOsM/L. When the toxins were dissolved at different molar concentrations in bath solution, it was supplemented with 0.1 mg/ml of BSA. The pipette filling (intracellular) solution consisted of 140 mM KF, 2 mM MgCl_2_, 1 mM CaCl_2_, 10 mM HEPES, and 11 mM EGTA, with a pH of 7.22 and an osmolarity of 295 mOsM/L.

To record the hKv1.3 and hKv1.2 currents, 15- to 200-ms-long depolarization impulses were applied at +50 mV from a holding potential of −120 mV every 15 s. The pClamp 10.1 software package was used to acquire and analyze the measured data. Current traces were low-pass filtered by the analog four-pole Bessel filters of the amplifiers, and the sampling frequency was set at 20 kHz, at least twice that of the filter cutoff frequency. The effect of the toxin at a given molar concentration was calculated as the remaining current fraction (RCF = I/I_0_, where I_0_ is the peak current in the absence of the toxin and I is the peak current at equilibrium block at a given toxin concentration). The data points on dose–response curves represent the mean of 3–4 individual measurements. The data points were fitted with a three-parameter [inhibition] vs response model, RCF = Bottom + [Top–Bottom)/(1+([toxin]/*K*_*d*_)], where Top and Bottom values were constrained to 1 and 0, respectively, and [toxin] is the concentration of the toxin. The best fit curve gave *K*_*d*_ of the given toxin.

### Analysis of the Binding and Unbinding Kinetics of the Peptides

To examine the effect of additional residues of TrMgTx on binding to the ion channel, the association and dissociation rate constants of both the versions of the toxin were determined for the Kv1.3 channel of the activated human T lymphocytes and the hKv1.2 channel transiently expressed in the CHO cell. Whole-cell currents were recorded after applying the toxin (200 pM for Kv1.3 and 100 pM for Kv1.2) to the extracellular solution until the equilibrium block was achieved (wash-in), and then removed it by perfusing the toxin-free control solution (washout). Peak currents at a time point t [I(t)] were normalized to the peak current in the absence of the toxin [I_norm_(t) = I(t)/I_0_], and these were plotted as a function of time. The association time constant (τ_*on*_) was determined by fitting the data points during the wash-in procedure in the one-phase decay equation: $I_{norm}\left(t\right)=(\left(1-RCF\right)\times e^{-\frac{t}{\tau_{on}}})+RCF$ , with the RCF = I/I_0_ at the equilibrium block as defined in the Electrophysiology section. The dissociation time constant (τ_*off*_) was determined by fitting the following equation to the data points during the washout procedure: $I_{norm}\left(t\right)=RCF+\left(1-e^{-\frac{t}{\tau_{off}}}\right).$

The association rate constant (*k*_*on*_) and dissociation rate constant (*k*_*off*_) were calculated from the measured time constants, assuming a simple bimolecular interaction between the channel and toxin, and by using the following equations (Peter et al., 2001), with τ_on_ and τ_off_ defined above, and [toxin] as the toxin concentration:

$$k_{on}=\frac{1-\left(\tau_{on}\times k_{off}\right)}{\tau_{on}\times \left[toxin\right]},k_{off}=\frac{1}{\tau_{off}}.$$

### IL2R and CD40L Expression Assay in CD4^+^ Effector Memory T Lymphocyte

The mononuclear cells were isolated from anonymized healthy donors as described earlier. Prior to CD4^+^ T_EM_ cells separation, dead cells were removed using the Dead Cell Removal Microbead Kit (Miltenyi Biotec B.V & CO. KG, Bergisch Gladbach, Germany). Untouched CD4^+^ T_EM_ lymphocytes were purified through magnetic cell sorting (negative selection) with the CD4^+^ Effector Memory T Cell Isolation Kit (Miltenyi Biotec B.V. & Co. KG, Bergisch Gladbach, Germany) according to the manufacturer's protocol.

For TCR-specific stimulation, anti-human CD3 monoclonal antibodies (clone OKT3, BioLegend, San Diego, CA) were bound to the surface of 24-well cell culture plates at a density of 5 µg/well in phosphate-buffered saline (PBS) overnight at 4°C. Before seeding the cells, the wells were washed twice with PBS to remove the unbound antibodies. The CD4^+^ T_EM_ cells were divided into four groups: 1) unstimulated, 2) stimulated, 3) stimulated + TrMgTx (8.5 nM), and 4) stimulated + UrMgTx (5 nM). The cells were seeded at a density of $0.5\times 10^{6}$ cells/ml per well, and when indicated, the cells were preincubated with the toxins for 5 min prior to the stimulation. The plate was incubated at 37°C in 5% CO_2_ for 24 h. Each experiment was performed in technical duplicates on three different donors.

For quantifying the extent of T_EM_ cell stimulation, cells were washed with PBS buffer containing 1% of fetal bovine serum (FBS) and stained with fluorescein isothiocyanate (FITC)–labeled anti-human CD154 (CD40L) antibody (clones 24–31, BioLegend, San Diego, CA) and PerCP/Cyanine5.5–labeled anti-human CD25 (IL2R) antibody (clone BC96, BioLegend) at 4°C for 20 min. Cells were washed with the PBS + 1% FBS buffer and resuspended in 150 µl in PBS + 1% FBS. Samples were measured with NovoCyte 3000 RYB flow cytometer (ACEA Bioscience Inc.), FITC and PerCP/Cyanine5.5 were excited by using blue laser (488 nm), and 530/30 nm and 695/40 nm emission filters were used, respectively. Flow cytometry data were analyzed using FCS Express 6.0 (De Novo Software, Glendale, CA). Briefly, cells were gated on the basis of their FSC and SSC parameters, and then, the histograms corresponding to CD40L and CD25 were plotted as peak-normalized overlays. Mean fluorescent intensities (MFIs) were computed from the histograms and normalized to that of their stimulated (S) but not treated control. The negative (unlabeled) and unstimulated control (US) were always used for comparison.

### Statistics

Data analyses and graph generation were performed using the GraphPad Prism software (version 9.1, La Jolla CA, United States). Statistical comparisons were made by using one-way ANOVA with Tukey's test and unpaired *t* test. For all the experiments, data are reported with standard error of mean (SEM).

## Results

### Transformation of TrMgTx–pPICZαA Recombinant Plasmid Into *P. pastoris* X-33 and Selection of Hyper-Resistant Transformants Against Zeocin

The expression cassette, consisting of six histidines followed by the factor Xa cleavage site and coding DNA sequence of MgTx (Figure 1B), was synthesized by using favorable codons for *P. pastoris* and cloned into pPICZαA expression vector under the control of alcohol oxidase I (AOX1) promoter. In-frame ligation to the α-factor secretion signal and nucleotide sequence of the TrMgTx was verified by DNA Sanger sequencing. A schematic illustration of the TrMgTx–pPICZαA recombinant plasmid was created by using the *in silico* SnapGene^®^ cloning tool (Figure 1A). The recombinant plasmid was linearized with the *SacI* enzyme and transformed into *Pichia* X-33 competent cells. Following 3 days of incubation at 30°C, more than 40 prominent colonies were observed on the YPD agar plate, containing 100 µg/ml of Zeocin. Hyper-resistant clones against Zeocin were selected by growing the initially chosen colonies on the YPD agar plates augmented with gradually increasing Zeocin to 0.5, 1, and 2 mg/ml (Supplementary Figure S1). The transformants that survived against the highest concentration of Zeocin (2 mg/ml) were selected, and colony PCR was performed which confirmed the integration of TrMgTx–pPICZαA plasmid by a single crossover at the 5′ AOX1 locus of the *P. pastoris* genome (Supplementary Figure S2).

### Overexpression of TrMgTx as a Function of Culturing Time, Methanol Induction, and pH of the Medium

**FIGURE 2:**
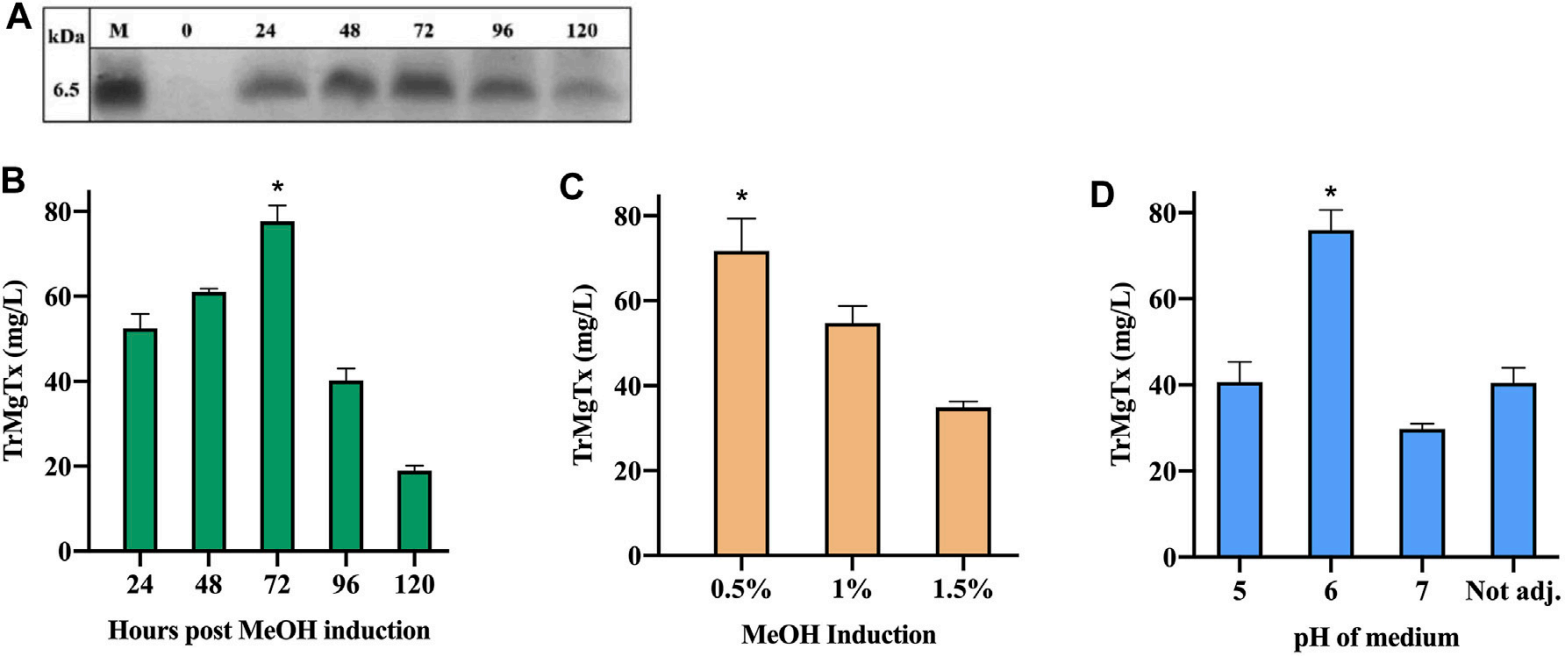
Optimization of TrMgTx expression in shake-flask fermentation. **(A)** 15 µl of supernatant samples were collected at indicated time points and analyzed by using 16% tricine–SDS-PAGE (stained with R-250 CBB). The samples from 24 to 120 h show bands of ∼6.5 kDa (close to the estimated MW of TrMgTx). **(B)** Concentration of TrMgTx in supernatant was quantified at the indicated time points by comparing the gel band intensity of TrMgTx with that of the standards by using Image Lab software (Bio-Rad). Expression was induced by 0.5% methanol at pH = 6.0 of the medium. **(C)** Secretion of TrMgTx in culture (at pH 6) induced with different MeOH concentrations for 72 h, peptide concentration was determined as in (**A**). **(D)** TrMgTx expression was determined as in **(A)** when induced with 0.5% MeOH for 72 h at the indicated pH values of the medium. Label “Not adj.” means that the pH of the medium was not buffered. Data represent the mean of three independent experiments, where the error bars are SEM. Asterisks indicate significant difference in all pairwise multiple comparison (Tukey's test), *p < 0.05.

Supernatant samples were collected from the cultures every 24 h post methanol induction for 5 days and were analyzed by tricine–SDS-PAGE. A peptide band of ∼6.5 kDa MW was detectable by using R-250 Coomassie Brilliant blue (CBB) after 24 h of methanol induction. This band gradually attained maximal density after 72 h of induction. However, the amount of secreted peptide toxin declined on the fourth and fifth day following induction (Figures 2A,B). The molecular mass of TrMgTx estimated from the gel was slightly higher than the predicted mass (5.9 kDa), most likely because of its higher PI value of 9.10. The highest concentration of peptide in the supernatant was 78 ± 7 mg/L after 72 h of induction. This yield was, in all pairwise multiple comparison (Tukey's test), significantly higher than the yield at any other time point (*p* < 0.05).

For optimization of methanol induction, biomass production of a TrMgTx clone was induced with 0.5, 1, and 1.5% MeOH, using a medium of pH 6. The gel scanning assay revealed that the amount of TrMgTx was the largest in the supernatant after inducing with 0.5% methanol for 72 h (71 ± 13 mg/L) (Figure 2C). This yield is significantly higher than that of the other two samples induced with 1 and 1.5% MeOH (all pairwise multiple comparison (Tukey's test), *p* < 0.05). Similarly, to find a suitable pH of the medium for improved expression of the peptide, biomass was induced with 0.5% MeOH in a media having pH 5, 6, or 7, or “not adjusted” for 72 h (Figure 2D). The supernatant of the culture at pH 6 showed 76 ± 8 mg/L expression of the peptide toxin which is considerably higher than for other samples from cultures with a pH 5 or 7 or “not adjusted” (all pairwise multiple comparison (Tukey's test), *p* < 0.05).

### Purification of Tagged Recombinant MgTx

**FIGURE 3:**
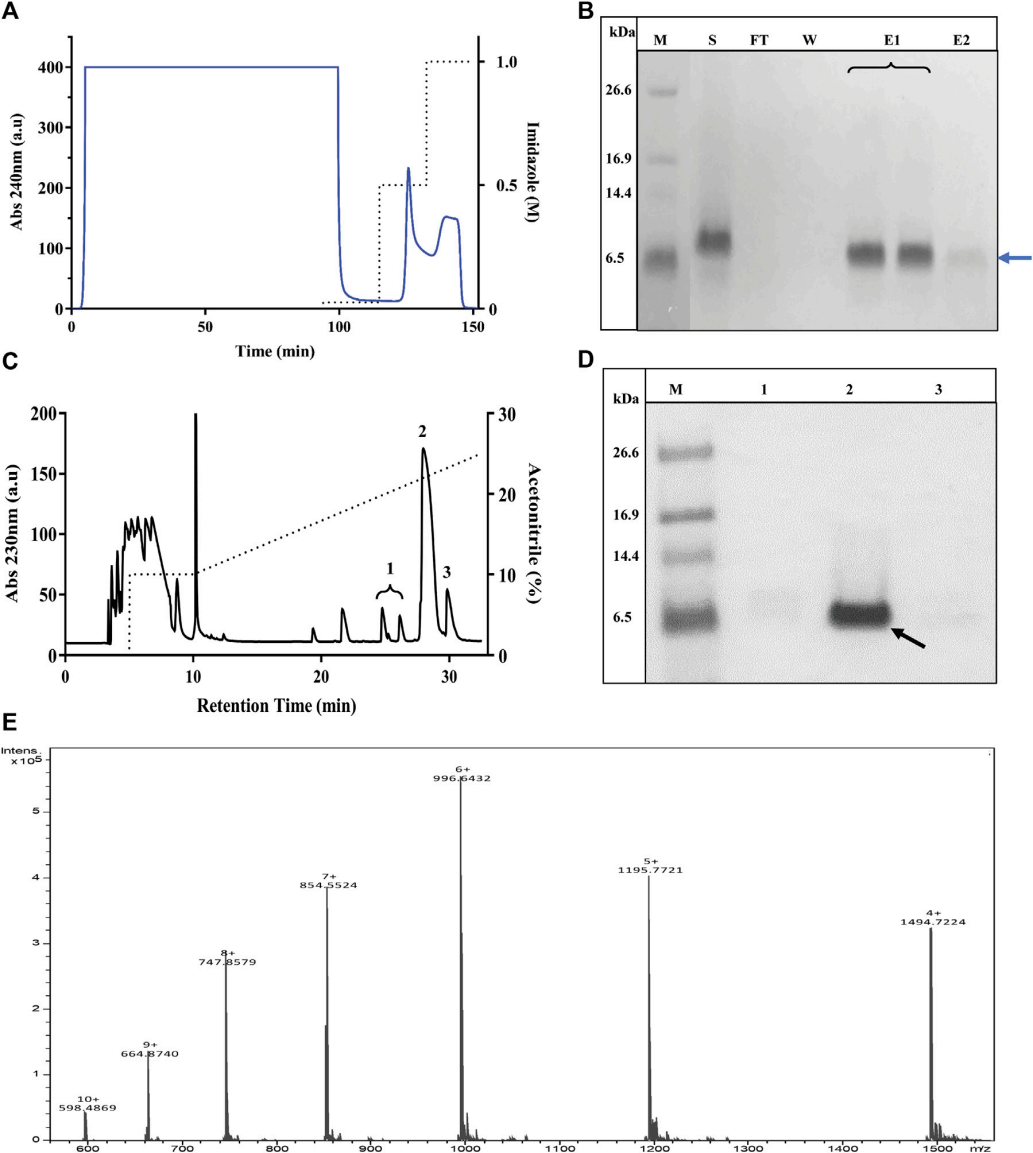
Purification of TrMgTx. **(A)** The chromatogram shows the loading of the supernatant of *P. pastoris* culture (cultured for 72 h, pH 6, induced with 0.5% MeOH) on a Ni^2+^ affinity column and elution with imidazole in isocratic mode. Absorbance was measured at 240 nm (indicated with blue line, left axis) and dotted line denotes the concentration of imidazole in elution buffer (right axis). **(B)** 16% Tricine–SDS-PAGE illustrates the analysis of fractions collected from Ni^2+^ affinity chromatography, where lane labels stand for **M**: low-molecular-weight (LMW) protein marker, **S**: raw (unpurified) supernatant, **FT**: flow through, **W**: wash with washing buffer, **E1**: elution with 0.5 M imidazole, and **E2**: elution with 1 M imidazole. A band at 6.5 kDa position (blue arrow) in lane E1 and E2 represents partially purified TrMgTx. **(C)** RP-HPLC chromatogram of TrMgTx. Partially purified TrMgTx in step 1 **(A,B)** was applied on RP-HPLC C_18_ semi-prep column and eluted with a gradient of 10–30% acetonitrile (shown with dotted line, right axis) over 30 min. Absorbance was recorded at 230 nm (left axis). Numbers indicate the peaks collected. **(D)** 16% Tricine–SDS-PAGE analysis of fractions collected from the RP-HPLC column. Lanes represent **M**: LMW protein marker, **1–3**: fractions from corresponding peaks as indicated in RP-HPLC chromatogram (panel **C**). The band of 6.5 kDa in lane 2 indicated with a black arrow represents purified TrMgTx. **(E)** ESI-QTOF-MS spectrum shows the average mass (5980.86 Da) of purified TrMgTx.

A *Pichia* X-33 clone that produced large amounts of TrMgTx in small cultures was subjected to large-scale fermentation in a medium of pH 6, and the expression was induced with 0.5% methanol for 3 days. The culture medium was centrifuged, filtered through a 0.45-µm membrane to remove cell debris, and then loaded on the Ni^+^ Sepharose column using the LC system as the first step of the two-step purification protocol applied. After removing the unbound proteins, His-tagged peptides were eluted with 0.5 and 1 M imidazole in isocratic mode. A very large peak appeared in the chromatogram with 0.5 M imidazole as compared with the peak obtained with 1 M imidazole, confirming that 0.5 M imidazole removed most of the bonded TrMgTx from the resin (Figure 3A). When the collected fractions were analyzed on tricine–SDS-PAGE, a clear and dense band of TrMgTx around ∼6.5 kDa was observed in the elution fraction (with 0.5 M imidazole) and a low-intensity band of the same size appeared in the fraction eluted with 1 M imidazole. On the contrary, no such band was observed in the fractions collected during the loading of the supernatant (flow though) and washing of the column (Figure 3B), demonstrating that resin had efficiently captured all His-tagged peptides from the cultured supernatant.

**FIGURE 4:**
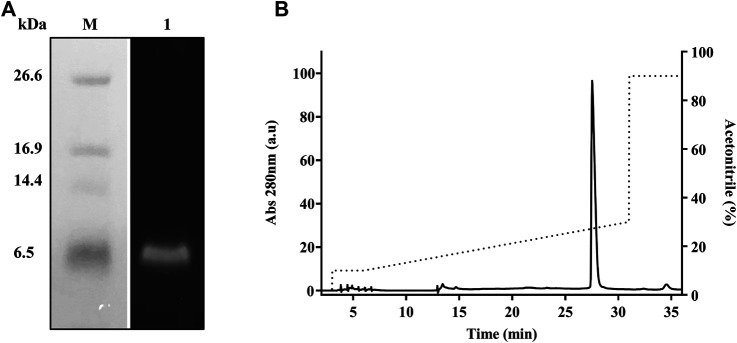
Purity analysis of TrMgTx produced by the two-step purification protocol. **(A)** Western blot of the sample eluted from the RP-HPLC column (Figures 3C,D) using HRP conjugated anti-His primary antibodies. **Lane M**: low-molecular-weight protein marker, **lane 1**: TrMgTx after RP-HPLC purification. **(B)** TrMgTx eluted from the RP-HPLC column (Figures 3C,D) was loaded on C_18_ analytical column and eluted with a gradient of 10–30% acetonitrile over 25 min (dotted line, right axis). The absorbance was measured at 280 nm (left axis). Single peak indicates TrMgTx. Purity was calculated as [(area under the peak of interest)/(cumulated area under all peaks) × 100] and is shown in Table 1.

To achieve high purity and homogeneity of the recombinantly produced TrMgTx, the partially purified fraction (i.e., eluted by 0.5 mM imidazole, see the Purification of Tagged Recombinant MgTx section for details) was applied on the C_18_ RP-HPLC semi-preparative column (second step of the purification protocol) and eluted with a gradient of 10–30% of acetonitrile in distilled water for 30 min (Figure 3C). Tricine–SDS-PAGE analyses of the collected HPLC fractions (Figure 3D) showed that a single band of TrMgTx at ∼6.5 kDa MW appeared in the fraction corresponding to the peak eluted at the retention time (R_T_) of ∼28 min (indicating to peak 2 in the chromatogram shown in Figure 3C). The average molecular mass of 5980.86 Da was determined for TrMgTx by ESI-QTOF-MS which is in full agreement with the predicted average mass (5980.96 Da) of the peptide (Figure 3E). The quality of the peptide after the two-step purification was assessed by using the anti-His antibody in the Western blot (Figure 4A) and HPLC (Figure 4B). The Western blot verified the purity and identity of TrMgTx by detecting a single band with anti-His antibodies (Figure 4A) of the appropriate size (cf. Figures 3B,D). The TrMgTx is more than 98% pure after the two-step purification as assessed by the C_18_ RP-HPLC analytical column (Figure 4B). Table 1 summarizes the purification scheme; on average, a total of 9.1 mg of TrMgTx was produced with 43% net recovery from 250 ml *P. pastoris* culture under optimized conditions.

**TABLE 1:** TrMgTx purification scheme.

	Purification Step	Avg. Vol. (ml)	Avg. TrMgTx Concentration (mg/ml)	Avg. Total TrMgTx Amount (mg)	^a^Avg. Net Recovery (%)	Purity (%)
–	Cultured supernatant	246	0.085^b^	20.8	100	–
1	Ni^2+^ affinity chromatography	14.5	1.01^b^	14.7	70.6	–
2	RP-HPLC**–**	32.6	0.279^c^	9.1	43.2	>98^d^

a Net recovery = TrMgTx acquired after a given step/total TrMgTx in cultured supernatant.

b Gel scan analysis with Image Lab Software (see Figure 2 legend).

c Pierce^TM^ BCA Protein Quantification kit.

d % Purity was assessed by RP-HPLC (see Figure 4B).

### Recombinant Margatoxin With Native N-Terminal

**FIGURE 5:**
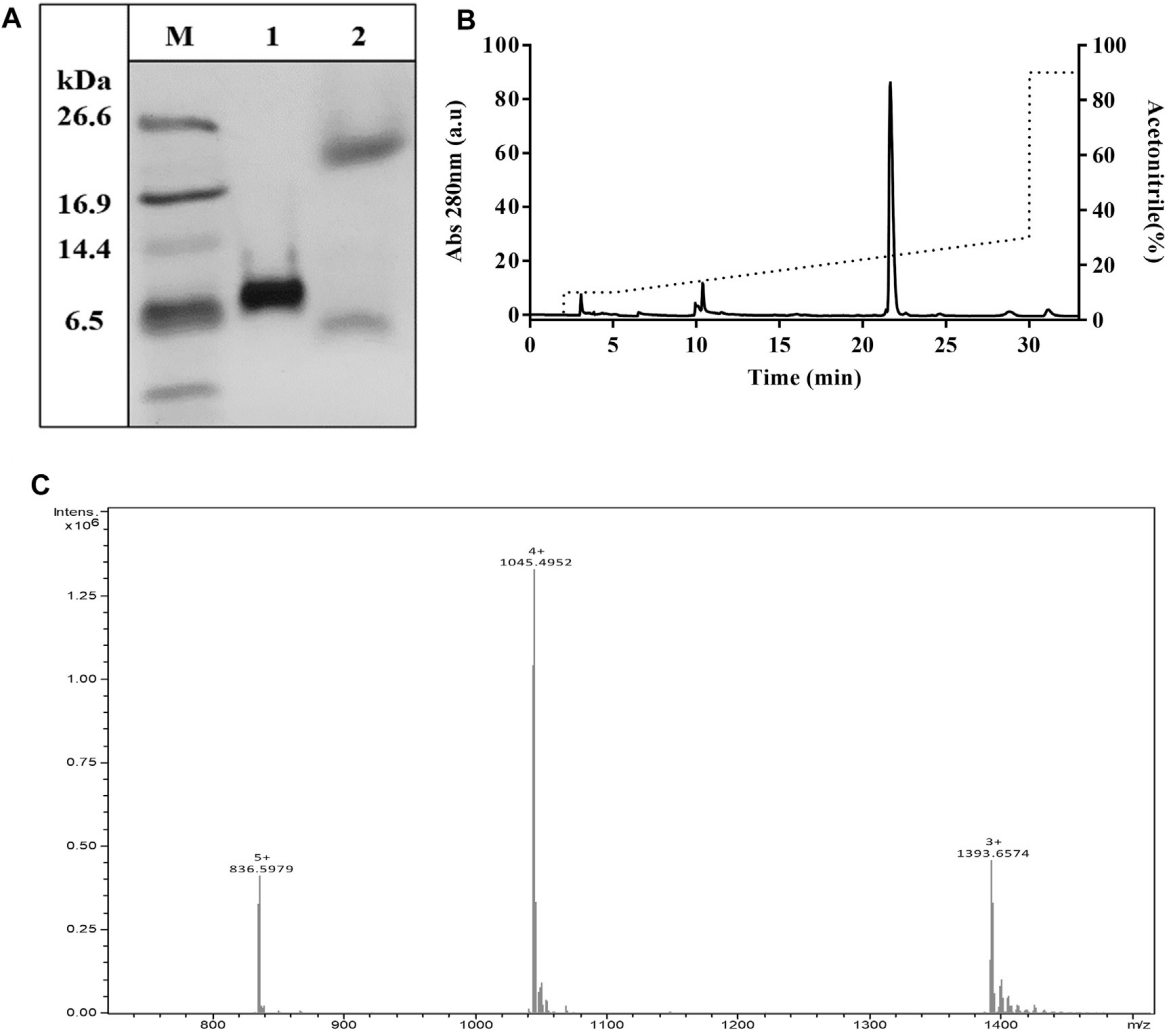
Removal of tag from TrMgTx. **(A)** 16% Tricine–SDS-PAGE analysis of the TrMgTx samples incubated overnight at 25°C without (**lane 1**) and with (**lane 2**) factor Xa protease (at 1:200 enzyme to peptide ratio); **M**: low-molecular-weight protein marker. **(B)** RP-HPLC chromatogram shows the purification of UrMgTx. After separating the His-tag fragments with Ni^+^ beads, the digested sample was loaded on C_18_ column and eluted with a gradient of 10–30% acetonitrile over 25 min. The absorbance was measured at 280 nm (left axis), dotted line shows the acetonitrile gradient (right axis). **(C)** ESI-QTOF-MS spectrum illustrates the average mass (4178.95 Da) of the purified UrMgTx.

TrMgTx contains 14 additional N-terminal amino acid residues (EFHHHHHHLQIEGR). This mainly consists of 6x histidines used for affinity purification and the protease cleavage site for factor Xa. Factor Xa protease cleaves the tagged peptides without leaving any extra amino acids at the N-terminal (Waugh, 2011). To get rMgTx with native N-terminal, all the additional residues were removed by digesting the tagged peptide with factor Xa protease overnight. Tricine–SDS-PAGE analyses (Figure 5A) revealed that a band of ∼4.1 kDa (the MW of native MgTx) was present in the overnight digested sample, confirming the successful removal of additional residues. The UrMgTx was purified using the C_18_ RP-HPLC column after separating the 6xHis fragments with Ni^+^ beads. The peak at R_T_ 21.6 min in the chromatogram (Figure 5B) indicates the elution of UrMgTx. The determined average mass (4178.95 Da) of UrMgTx is equivalent to the theoretical mass (4179.018 Da) of native MgTx, proving that there is no extra residue at either terminal of UrMgTx after cleaving the tag (Figure 5C).

### Effect of Tagged and Untagged Recombinant Margatoxin on hKv1.2 and hKv1.3 Ion Channel

Pharmacological activity of both versions of rMgTx was evaluated by studying their effect on the hKv1.2 and hKv1.3 ion channels. The hKv1.2 current was recorded in CHO cells, which heterologously expressed the hKv1.2 ion channel, and hKv1.3 current was recorded in the human peripheral blood lymphocytes. The stimulation of the hKv1.3 channel expression (activation of isolated mononuclear cells by PHA, see the Materials and Methods section) and recording conditions (no Ca^2+^ in the pipette to elicit Ca^2+^-activated K^+^ channels) guaranteed that the current recorded in these cells is K^+^ current through Kv1.3 (Varga et al., 2012; Bartok et al., 2014; Tajti et al., 2021). A custom-built perfusion system was used to apply toxins at a very small perfusion rate of 200 µl/min. The speed and complete solution exchange in the recording chamber at this perfusion rate were tested frequently using fully reversible blockers as a positive control. Around 50% reduction in the peak current upon perfusion of the positive control in concentrations equal to the respective *K*_*d*_ values, i.e., 10 mM tetraethylammonium for hKv1.3 and 14 nM charybdotoxin for hKv1.2, proved the complete exchange of solution (data not shown).

**FIGURE 6:**
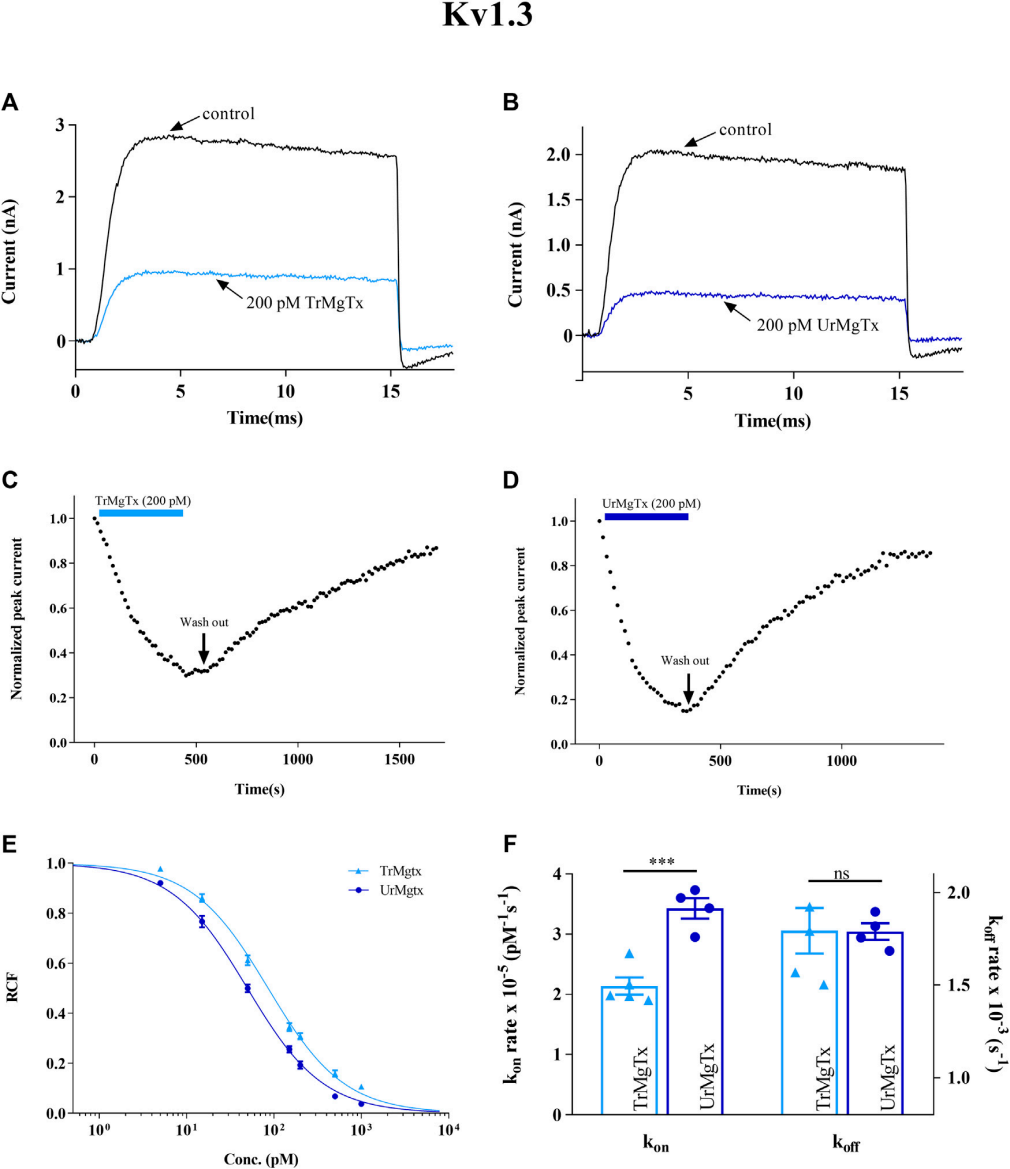
Inhibition of hKv1.3 currents by tagged and untagged recombinant MgTx. **(A,B)** Whole-cell currents through hKv1.3 were evoked from activated human peripheral lymphocytes by depolarization to +50 mV from a holding potential −120 mV for 15 ms duration. Test pulses were applied every 15 s. Representative traces show the K^+^ current before the application of toxin (control) and after reaching the equilibrium block in the presence TrMgTx **(A)** or UrMgTx **(B)** at 200 pM concentration (as indicated). **(C,D)** Development of and recovery from the block of hKv1.3 current. Normalized peak currents were measured in whole-cell patch configuration and plotted against time as 200 pM of TrMgTx [**(C)**, light blue bar] or UrMgTx [**(D)**, dark blue bar] was applied to the bath solution. Following block equilibrium, the recording chamber was perfused with toxin-free solution (arrow, washout) to demonstrate reversibility of the block. **(E)** Concentration-dependent block of hKv1.3 by TrMgTx and UrMgTx. The RCF values taken at different toxin concentrations were fitted with a three-parameter (inhibition) vs response model (see the Materials and Methods section for details). The best fit resulted in *K*_*d*_ = 50 pM for UrMgTx and *K*_*d*_ = 86 pM for TrMgTx. Error bar represents SEM and *n* = 3–4. **(F)** Comparison of block kinetics of TrMgTx and UrMgTx for hKv1.3. Association rate constant *k*_*on*_ (left y-axis) and dissociation rate constant *k*_*off*_ (right y-axis) were calculated from measured time constants (τ_*on,*_ τ_*off*_) for the development of the block in the presence of 200 pM toxin and the washout (see panel **C**,**D**), assuming a simple bimolecular reaction between the channel and toxin (see the Materials and Methods section for details) and plotted on bar graph. Symbols indicate individual data points (*n* = 4); bar heights and error bars indicate mean ± SEM. ***p < 0.001, ns = not significant, unpaired *t* test.

TrMgTx and UrMgTx at the 200 pM concentration inhibited ∼70 and ∼80% of the whole-cell hKv1.3 current upon reaching the block equilibrium, respectively. Figures 6A,B show the current traces recorded in the presence and absence of the respective peptide toxins. Figures 6C,D demonstrate the development and recovery from the block of the hK1.3 current with TrMgTx and UrMgTx as indicated using the colored bars. In case of hKv1.3, the block equilibrium develops slower for TrMgTx than for UrMgTx upon application of 200 pM of toxin in bath solution, as reported by the time constants (τ_*on*_) obtained by fitting a single exponential function to the decay of the peak currents in the presence of the blockers (for TrMgTx *τ*_*on*_ = 168 ± 19 s and for UrMgTx *τ*_*on*_ = 116 ± 10 s were obtained, *n* = 4). Almost full recovery of the peak currents was attained upon perfusing the bath medium with toxin-free solution with similar time constants for washout (τ_*off*_) for the two peptides (for TrMgTx *τ*_*off*_ = 568 ± 84 s and for UrMgTx *τ*_*off*_ = 560 ± 28 s were obtained, *n* = 4). To reveal the impact of additional amino acids at the N-terminal of TrMgTx on the interaction with hKv1.3, blocking parameters *k*_*on*_, *k*_*off*_, and equilibrium dissociation constant *K*_*d*_ were calculated (Table 2) from the measured time constant values and plotted on the bar graph in Figure 6F. The *k*_*on*_ rate of UrMgTx was significantly higher than was for TrMgTx (in unpaired *t test* comparison, *n* = 4, *p* < 0.001); however, the *k*_*off*_ rate of the peptides was statistically the same (*p* > 0.05, *n* = 4). The hKv1.3 blocking potency of both tagged and UrMgTx was obtained by determining their *k*_*d*_ values by using dose–response relationships as well. The data points were fitted using a three-parameter [inhibition] vs response equation, and the best fit gave the *k*_*d*_ 50 and 86 pM for UrMgTx and TrMgTx, respectively, as is shown in Figure 6E. Based on the dose–response relationship, the tagged version of the toxin, TrMgTx, is slightly less potent for hKv1.3 than the tag-free version of the peptide, UrMgTx.

**TABLE 2:** Kinetic parameters of rMgTx and Kv channel interaction.

Kv Channel	Toxin	*k*_*on*_ (pM^−1^s^−1^)	*k*_*off*_ (sec^−1^)	*K*_*d*_ (pM)
Kv1.3	TrMgTx	2.14E-05	0.00179	83.90
	UrMgTx	3.43E-05	0.00179	52.20
Kv1.2	TrMgTx	3.05E-05	0.00190	62.29
	UrMgTx	4.62E-05	0.00098	15.04

*k*_*on*_ and *k*_*off*_ were calculated from averaged τ_*on*_ and τ_*off*_ values obtained from 3–5 independent experiments (see the Materials and Methods section for details). *K*_*d*_ was determined as *k*_*off*_/*k*_*on*_.

**FIGURE 7:**
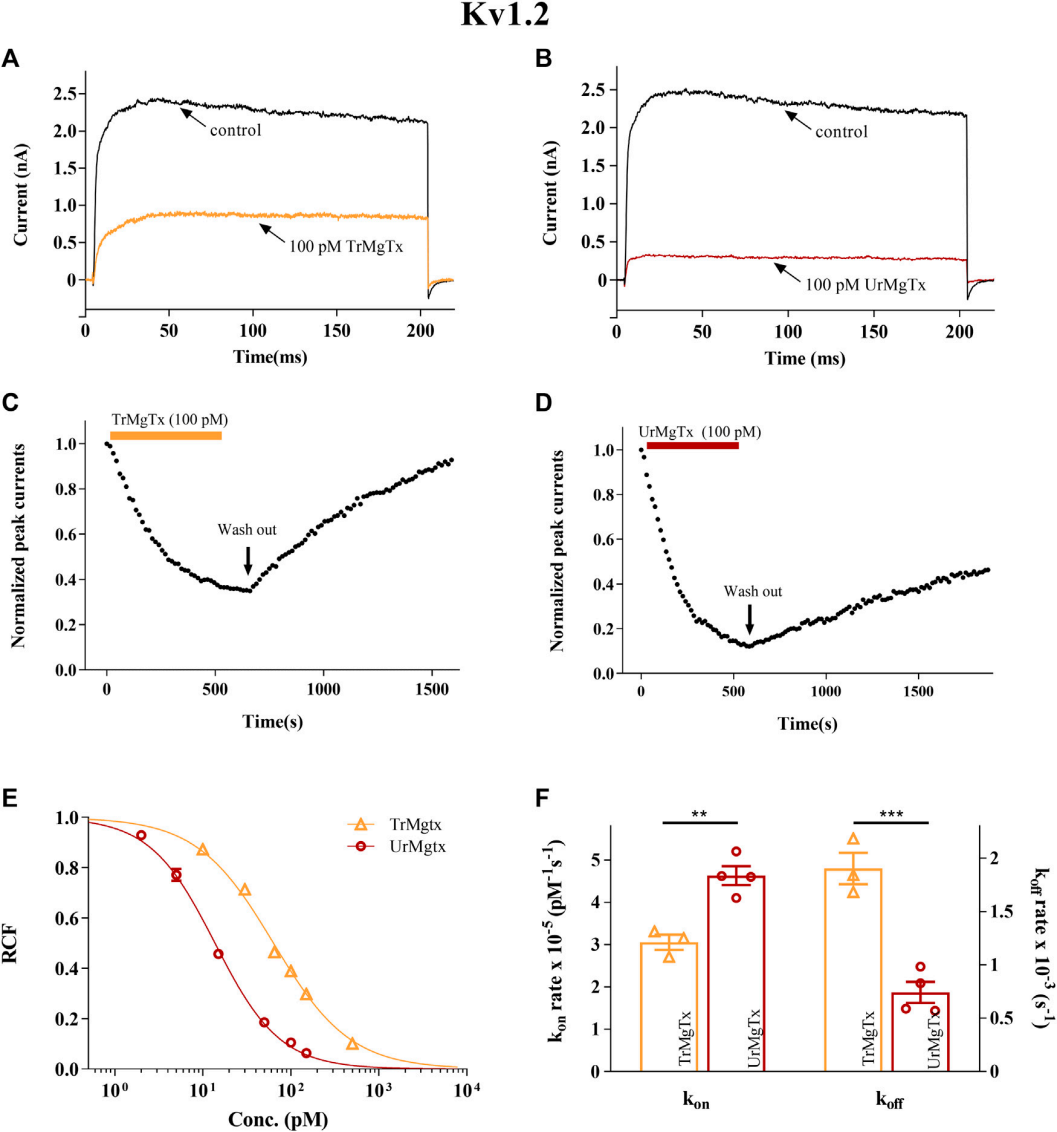
Inhibition of hKv1.2 currents by tagged and untagged recombinant MgTx. **(A,B)** The K^+^ currents through hKv1.2 were evoked from transiently transfected CHO cells either in whole-cell or outside-out patch configuration by depolarization to +50 mV from a holding potential −120 mV for 200 ms duration. Test pulses were applied every 15 s. Representative traces show the K^+^ current before the application of toxin (control) and after reaching the equilibrium block in the presence TrMgTx **(A)** or UrMgTx **(B)** in 100 pM concentration (as indicated). **(C,D)** Development of and recovery from block of hKv1.2 current. Normalized peak currents were measured in whole-cell patch configuration and plotted against time as 100 pM of either TrMgTx (C, yellow bar) or UrMgTx (D, burgundy bar) was applied to the bath solution. Following block equilibrium, the recording chamber was perfused with toxin-free solution (arrow, washout) to assess reversibility of the block. **(E)** Concentration-dependent block of hKv1.2 by TrMgTx and UrMgTx. The RCF values taken at different toxin concentrations were fitted with a three-parameter (inhibition) vs response model (see the Materials and Methods section for details). The best fit yielded *K*_*d*_ = 14 pM for UrMgTx and *K*_*d*_ = 64 pM for TrMgTx. Error bars represent SEM and *n* = 3–5. **(F)** Comparison of block kinetics of TrMgTx and UrMgTx for hKv1.2. Association rate constant *k*_*on*_ (left y-axis) and dissociation rate constant *k*_*off*_ (right y-axis) were calculated from measured time constants (τ_*on*_, τ_*off*_) for the development of the block in the presence of 100 pM toxin and the washout (see **C** and **D**), assuming a simple bimolecular reaction between the channel and toxin (see the Materials and Methods section for details) and plotted on bar graph. Symbols indicate individual data points (*n* = 3–4), bar heights and error bars indicate mean ± SEM. **p < 0.01, ***p < 0.001, ns = not significant, unpaired *t* test comparison.

A similar set of experiments were repeated for the hKv1.2 ion channel expressed in CHO cells. TrMgTx and UrMgTx at 100 pM concentrations blocked about 60 and 90% of the whole-cell current of hK1.2, respectively, as shown by the current traces recorded in the presence and absence of the respective peptide toxins (Figures 7A,B). The change in the peak current of hK1.2 upon application and washout of the TrMgTx and UrMgTx (colored bars) is shown in Figures 7C,D. Like hKv1.3, the equilibrium block of hKv1.2 with TrMgTx developed on a slower time course than with the UrMgTx at identical (100 pM) concentrations (for TrMgTx *τ*_*on*_ = 202 ± 2.3 s and for UrMgTx *τ*_*on*_ = 191 ± 12 s). Perfusion of the bath with toxin-free solution results in slow but full recovery (*τ*_*off*_ = 532 ± 69 s) from the block in case of TrMgTx. On the contrary, in case of UrMgTx, slow and partial recovery (*τ*_*off*_ = 1308 ± 351 s) from the block was observed. Blocking parameters *k*_*on*_, *k*_*off*_, and *K*_*d*_ for hKv1.2 were calculated from measured time constant values as given in Table 2 and plotted on a bar graph (Figure 7F). The *k*_*on*_ obtained for UrMgTx is slightly higher than that of TrMgTx (unpaired *t test, n* = 3−4, *p* < 0.01); however, the *k*_*off*_ rate of UrMgTx is substantially lower than that of TrMgTx (unpaired *t test*, *n* = 3−4, *p* < 0.001). In the dose–response relationship, the best fit of data points results in *K*_*d*_ = 64 pM for TrMgTx and *K*_*d*_ = 14 pM for UrMgTx (Figure 7E). Tagged TrMgTx is nearly fivefold less potent for hKv1.2 than tag-free UrMgTx.

### Kv1.3 Inhibition by rMgTx Decreases the Activation of CD4^+^ T_EM_ Cells

**FIGURE 8:**
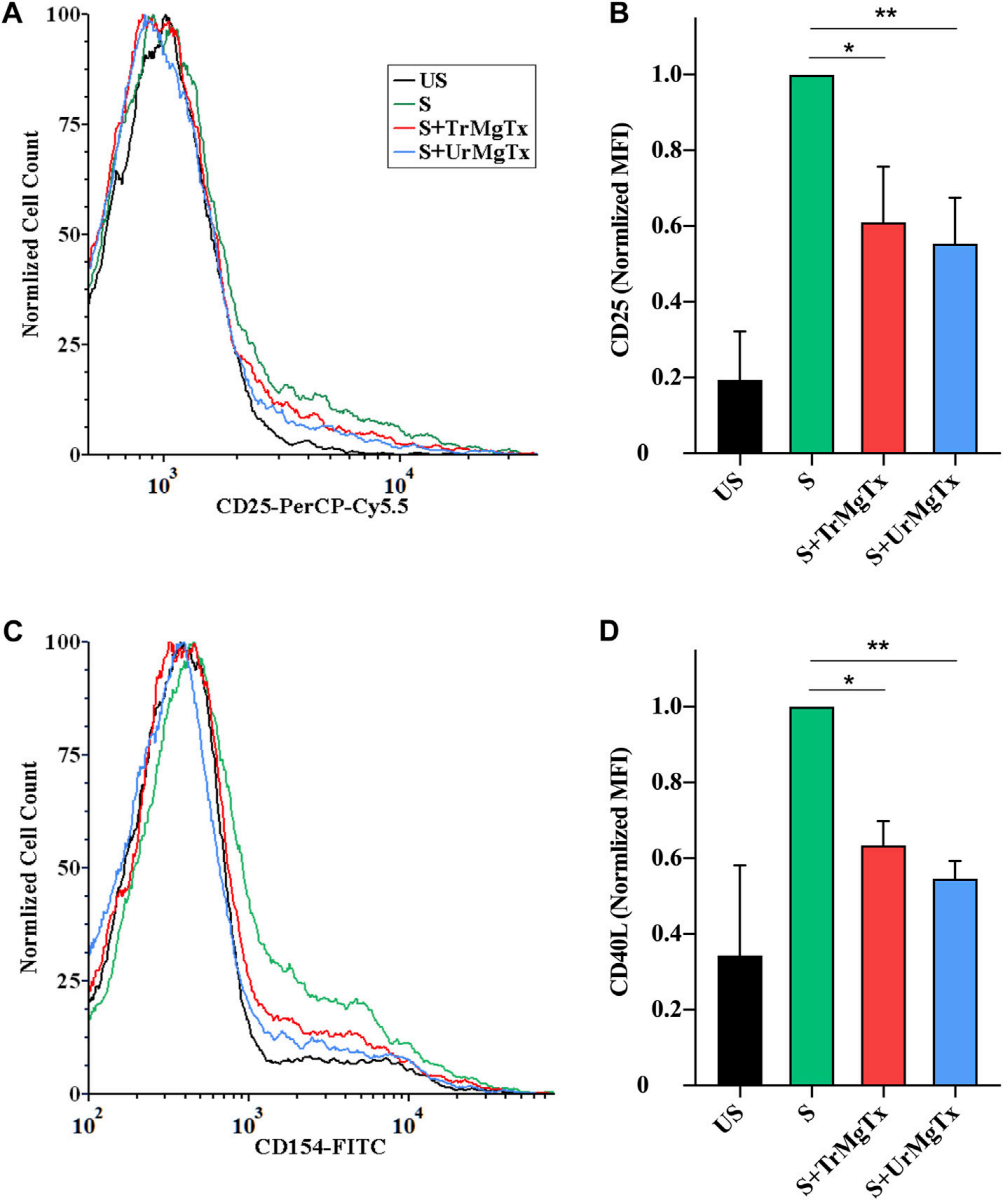
rMgTx decreases the expression of IL2R (CD25) and CD40L. Isolated CD4^+^ T_EM_ cells were stimulated with anti-human CD3 antibody in the presence or absence of toxins. After 24 h of stimulation, cells were labelled with anti-CD25 (IL2R) **(A,B)** and anti-CD154 (CD40L) antibodies **(C,D)**. Treatment labels: US, unstimulated (black); S, stimulated with anti-human CD3 antibody–coated wells **(**5 µg/well; see the Materials and Methods section for details, green); S + TrMgTx, stimulated in the presence of TrMgTx (8.5 nM, red); S + UrMgTx, stimulated in the presence of UrMgTx (5 nM, blue). **(A,C)** Fluorescence histograms were obtained from T lymphocytes gated based on their FSC and SSC parameters (10,000 events were recorded), and then, histograms corresponding to CD25 or CD154 expression were plotted as peak-normalized overlay. Plots were made using FCS Express 6.0. (**A,C**) has the same color code. **(B,D)** Mean fluorescence intensities (MFI) were determined from the histograms and normalized to that of their stimulated but not treated control (S). Data represent values from three independent experiments (two technical repeats in each) with SEM. Significant differences of IL2R and CD40L expression between the stimulated samples in the absence and presence of toxin is indicated with asterisks (*p < 0.05, **p < 0.01, all pairwise multiple comparison).

Upon T lymphocyte stimulation, the expression of activation markers in the cell membrane, such as IL2R and CD40 ligand, are upregulated. High-affinity blockers of Kv1.3 inhibit the activation and proliferation of human T_EM_ cells through decreasing the driving force for the Ca^2+^ influx (Chandy et al., 2004; Cahalan and Chandy, 2009). In this study, it has been investigated whether recombinantly produced MgTx (with tag or without tag) would affect the expression of IL2R and CD40L, as activation markers in CD4^+^ T_EM_ cells upon TCR ligation for 24 h. The presence of either toxin TrMgTx or UrMgTx (at ∼100× concentration of their respective *K*_*d*_ values) during the T_EM_ cell stimulation significantly reduced the upregulation of IL2R and CD40L expression. About 8.5 nM of TrMgTx showed ∼39% inhibition of IL2R expression and ∼36% inhibition of CD40L expression. Similarly, 5 nM of UrMgTx hampered expression of both activation markers by ∼45% (Figures 8A-D). It is important to note that CD40L expression of the stimulated cells after treatment with either TrMgTx or UrMgTx was comparable to that of unstimulated cells (Figure 8D). These results suggest that both TrMgTx and UrMgTx are biologically active peptides; however, TrMgTx has a slightly lesser efficacy than UrMgTx due to the presence of a His-tag at the N-terminus.

## Discussion

In this article, an improved production of rMgTx in the *P. pastoris* expression system has been described by optimizing various aspects of the vector and the selection/culturing conditions (pH, methanol induction, and fermentation duration) and confirmed the applicability of the recombinant peptides in electrophysiological and functional assays. Firstly, the coding DNA sequence of MgTx was generated by using preferred codons for *P. pastoris*, and Zeocin (2 mg/ml) hyper-resistant transformants were selected for expression studies. Secondly, upon substantial optimization, a yield of 83 ± 3 mg/L TrMgTx (i.e., His-tagged rMgTx) was obtained in the culture supernatant at pH 6.0 of the culturing medium and 72 h of fermentation following 0.5% methanol induction. Following Ni^2+^ affinity chromatography/RP-HPLC purification, the final yield of 36 ± 4 mg/L TrMgTx was obtained at >98% purity, as confirmed by RP-HPLC, mass spectrometry, and Western blot analysis, using anti-His antibody. Thirdly, to compare the potency of TrMgTx in inhibiting Kv1.2 and Kv1.3 channels with that of the untagged rMgTx (i.e., UrMgTx) having native-like N-terminal, TrMgTx was digested using factor Xa protease, and subsequently purified with RP-HPLC, and the cleavage was confirmed using mass spectrometry. In the electrophysiology assay (patch-clamp), it was shown in this study that TrMgTx is slightly less potent than UrMgTx in inhibition of either Kv1.2 (*K*_*d*_ = 64 vs 14 pM) or Kv1.3 (*K*_*d*_ = 86 vs 50 pM), albeit both peptides displayed picomolar affinity for the channels. The analysis of the association and dissociation kinetics showed that the somewhat lower affinity of TrMgTx for Kv1.2 can be explained by the lower association and increased dissociation rate of the toxin–channel complex as compared with UrMgTx. Fourthly, in a biological functional study, it was shown that both peptides significantly reduced the expression of IL2R and CD40L in activated CD4^+^ T_EM_ cells at a 100× higher concentration than the *K*_*d*_ values of the respective toxins for Kv1.3.

Scorpion venom–derived peptide toxins have been under the spotlight for the past 2 decades, especially α-KTxs, as they effectively block the Kv1.3 channel in sub-nanomolar concentrations. Therefore, these can be used to treat autoimmune diseases through blockade of upregulated Kv1.3 channel in autoreactive human CD4^+^ T_EM_ cells (Shen et al., 2017; Tajti et al., 2020). However, selectivity of these peptide toxins for Kv1.3 is still an issue because they block various other subtypes of Kv ion channels with high affinity (Kalman et al., 1998; Bagdány et al., 2005). Consequently, these undesired effects may restrict their use as potential therapeutic drugs. Selectivity of these peptides for Kv1.3 can be enhanced by peptide engineering. One example is the double substituted analog of Anuroctoxin that exhibits more than 16,000-fold selectivity for Kv1.3 over Kv1.2 (as compared with the 10-fold selectivity of the wild-type toxin), while maintaining the affinity towards Kv1.3 (Bartok et al., 2015). Similarly, the Kv1.3 selectivity of HsTx1 over Kv1.1 was improved significantly by R14A substitution (Rashid et al., 2014). In addition, peptide toxins are widely used to investigate the function of Kv1.x ion channels in various cell types *in vivo* as well as *in vitro* (Erdogan et al., 2005; Tubert et al., 2016; Schwartz et al., 2020; Toldi et al., 2020). Obviously, one needs a reliable and economical system to produce an ample amount of these peptide toxins and their analogs to develop them as therapeutic drugs for the treatment of autoimmune disorders and to study the physiological role of ion channels.

The natural resources (e.g., venom glands) provide a very minute quantity of pure wild-type peptides, which is usually insufficient for structural and functional experiments. Since peptide toxins consist of 30–80 amino acids and they maintain their tertiary structure with 3–4 disulfide bonds, chemical synthesis (Jensen et al., 2009) and recombinant production in *E. coli* of such peptides need oxidative refolding and repurification to get the correctly refolded and biological active isomer (Rudolph and Lilie, 1996). Some engineered *E. coli* strains, on the other hand, are capable of disulfide bond formation and refold the peptide (Lobstein et al., 2012; Klint et al., 2013). However, neither chemical synthesis nor *E. coli* recombinant production approaches seem to be efficient to produce a high yield of disulfide-rich peptides. For example, MgTx was previously produced in *E. coli* yielding 3–4 mg/L of functional peptides (Garcia-Calvo et al., 1993). The *P. pastoris* expression system offers an economical and better approach to overcome these limitations and gives higher yields of correctly folded recombinant peptides. This system has many pros as a host for heterologous production of proteins, such as high biomass production in simple medium, ease of genetic manipulation, and capability of performing posttranslational modifications. Additionally, *P. pastoris* secretes the heterologous proteins into medium with very few endogenous proteins which significantly simplifies the downstream processing (Macauley-Patrick et al., 2005; Cregg, 2007). In line with these, Anangi et al. (2012) expressed and purified the His-tagged MgTx and Agitoxin in the *Pichia* expression system with a yield of 12–15 mg/L and 14–18 mg/L, respectively (Anangi et al., 2012).

The expression level of heterologous proteins in *P. pastoris* can be enhanced by adopting various strategies. These include the usage of codon-optimized genes, screening for high-copy integrant, choice of efficient promoter, and optimization of cell fermentation conditions (Macauley-Patrick et al., 2005; Yu et al., 2010). Previous studies report that the use of codon-optimized gene sequence increases the heterologous protein expression about 1- to 10-fold in *P. pastoris* over the native gene sequence (Yadava and Ockenhouse, 2003; Li et al., 2010; Yu et al., 2013; Wang et al., 2015). For example, codon-optimized glucanase gene from *B. licheniformis* was expressed 10-fold higher compared with the wild-type gene in *P. pastoris* (Teng et al., 2007). To improve the recombinant expression, the codon-biased MgTx gene was generated according to the codon usage table for *P. pastoris* available at www.kazusa.or.jp/codon. Generally, it has also been observed that an increase in the copy number of the expression cassette significantly increases the expression of the target gene (Clare et al., 1991; Vassileva et al., 2001; Mansur et al., 2005; Nordén et al., 2011). Upon transformation into *P. pastoris*, multicopy integration of expression cassette could occur spontaneously at single locus by homologous recombination. In this study, multicopy clones of *P. pastoris* were created by using the post-transformational vector amplification method described by Sunga et al. (2008). Initially, transformants were selected on low concentration of Zeocin and then subject to progressively increasing higher concentrations (up to 2 mg/ml of Zeocin). Nordén et al. (2011) reported that hyper-resistance against Zeocin was intimately correlated with enhanced expression of foreign proteins in *P. pastoris*. As such, this strategy was followed to select hyper-resistant *P. pastoris* strains.

The expression level of recombinant proteins in *P. pastoris* is a function of several fermentation factors such as biomass production, pH of the medium, methanol induction, dissolved oxygen, and medium types. The influence of these factors on heterologous protein expression in *P. pastoris* varies for each protein (Macauley-Patrick et al., 2005; André et al., 2006; Khatri and Hoffmann, 2006; Minjie and Zhongping, 2013). Thus, in the present study, to achieve maximal expression of rMgTx, first the time course of methanol-induced peptide production was optimized and later the optimal concentration of methanol for induction and the optimal pH of the medium were determined. As a strong methanol-induced promoter, *AOX1* was employed here, and different concentrations of methanol were expected to significantly alter the heterologous protein expressions. Based on various foreign proteins, the suitable methanol concentration ranged between 0.1 and 3% (v/v) (Minjie and Zhongping, 2013). When the biomass was induced with 0.5% methanol in BMMY medium for 72 h, it was found to yield the highest titer of TrMgTx. Similarly, Mu et al. (2008) reported that the highest concentration of basic fibroblast growth factor was obtained at 0.5% (v/v) MeOH and yielded 150 mg/L peptide after 72 h of induction. On the contrary, Zhang et al. (2009) suggested that moderate methanol concentration and induction time to get the maximal expression of inulinases in *P. pastoris* were 1.5% (v/v) and 72 h, respectively. The pH of the medium is also an essential factor to be optimized to improve the titer of heterologous protein in *P. pastoris* because the stability of foreign protein depends on the pH of the culturing medium*. P. pastoris* can grow in a wide pH range of 3.0–7.0, without altering the growth rate drastically; however, the optimal pH varies for different recombinant proteins (Macauley-Patrick et al., 2005; Minjie and Zhongping, 2013). In this study, the highest level of rMgTx expression in culturing medium with pH 6.0 was observed. Likewise, studies have reported that the optimal pH for production of mouse epidermal growth factor, α-amylase, and human serum albumin in *P. pastoris* is pH 6.0 as well (Kobayashi et al., 2000; Park, 2006), whereas the optimal pH for the human growth hormone and insulin-like growth factor-I are 5.0 and 3.0, respectively (Brierley et al., 1994; Çalık et al., 2010). These results indicate that methanol induction and pH, among others, must be optimized for individual proteins. Conclusively, the highest concentration of TrMgTx in the culture medium was 83 ± 3 mg/L when the *P. pastoris* clone was induced under optimized conditions (0.5% MeOH, pH 6.0 for 72 h). The two-step purification strategy exploited in this study resulted in more than 98% pure peptide, albeit the final yield reduced to 36 ± 4 mg of TrMgTx from 1 L of culture. The final yield is threefold higher than previously reported yields for TrMgTx in *P. pastoris* and is 10–12 times higher than the production in *E. coli* (Garcia-Calvo et al., 1993; Johnson et al., 1994; Anangi et al., 2012).

In this pharmacological study using patch-clamp electrophysiology, it has been demonstrated that both versions of recombinantly produced MgTx (tagged and untagged) potently blocked hKv1.3 and hKv1.2 currents. The *K*_*d*_ values of the UrMgTx for hKv1.2 and hKv1.3 channels are in good agreement with the previous data on the native or rMgTx (Garcia-Calvo et al., 1993; Chandy et al., 2004; Bartok et al., 2014). However, the affinity of TrMgTx for both hK1.3 and hK1.2 was slightly less than that for UrMgTx. The reduced affinity of TrMgTx may be related to the additional 14 residues at its N-terminal that may interfere with the interaction of TrMgTx with the pore region of the ion channel. Chang et al. (2015) demonstrated that N-terminally extended analogs of Shk toxin inhibited the hKv1.3 channel with reduced potency as compared with the native peptide toxin.

The kinetics of current block by UrMgTx was consistent with previously reported data for MgTx. In case of hKv1.3, the equilibrium block develops rapidly and is fully recoverable upon washout, whereas the block of hKv1.2 develops relatively slowly and reversibility of the block is limited during the time scale of the whole-cell patch-clamp recording (Bartok et al., 2014). The association rate of TrMgTx, which has the N-terminal His-tag, was ∼1.6-fold lower than that of UrMgTx for both hKv1.2 and hKv1.3. The reduced *k*_*on*_ rate could be explained by non–diffusion-limited bimolecular toxin–channel interactions described by Escobar et al. (1993) and Peter et al. (2001). In this model, the transition from the toxin–channel encounter complex to the toxin bound state (B) is a rate-limiting step for the block of pores of the channels. This transition may be sensitive to the rearrangement of amino acid side chains, hydrogen-bond formation, and squeezing water molecules and cations out of the extracellular vestibule of the channel to make stable toxin–channel complex (Peter et al., 2001). This process might have been altered by the additional amino acids at the N-terminus of tagged toxin, thereby leading to the decrease in *k*_*on*_ observed in this study. On the other hand, the dissociation rate (*k*_*off*_) of TrMgTx was identical to that of UrMgTx for hKv1.3, but *k*_*off*_ was much higher for TrMgTx than for UrMgTx in case of hKv1.2. Moreover, the block of Kv1.2 by TrMgTx was fully reversible upon washout that is in clear contrast to UrMgTx. The increased *k*_*off*_ might indicate that the presence of the N-terminal tag in TrMgTx creates unfavorable interactions between the channel and the bound peptide, thereby shortening the lifetime of the toxin bound channel (Peter et al., 2001). Although TrMgTx is slightly less potent than UrMgTx with different block kinetics, the TrMgTx can be an excellent tool, as 1) it inhibits hKv1.2 and hKv1.3 in picomolar concentrations, 2) the reversible nature of the block of hKv1.2 might be preferable for the physiological study of ion channels, and 3) the production of TrMgTx does not require proteolytic cleavage of tag and downstream isolation steps, which makes the production straightforward, easy, and cost-efficient. Moreover, the His-tag of TrMgTx can be recognized by anti–His-tag antibodies and can potentially be used to determine the toxin concertation in biological fluids (Borrego et al., *manuscript in preparation*).

Kv1.3 expression is dramatically upregulated in effector memory T (T_EM_) cells upon activation (Wulff et al., 2003). Inhibition of Kv1.3 by high-affinity blockers impede the Ca^+^-dependent response to TCR activation and subsequently cell proliferation (Cahalan and Chandy, 2009). It was demonstrated in this study that the TrMgTx and UrMgTx strongly inhibited the expression of the early activation markers interleukin-2 receptor α (CD25) and CD40 ligand in anti–CD3-activated CD4^+^ T_EM_ lymphocytes, presumably due to the lack of calcineurin activation and the consequent translocation of NFAT to the CD25 and CD40L promoters (Tsytsykova et al., 1996; Schuh et al., 1998). Approximately 100× higher toxin concentrations than the *K*_*d*_ of the respective peptides for Kv1.3 were used in this assay to ensure practically a complete blockade of the Kv1.3 channels. The use of high toxin concentrations is in accordance with previous reports where significantly higher concentrations of Vm24 and Shk were used in biological assays than the *K*_*d*_ of the toxin for Kv1.3 (Beeton et al., 2011a; Veytia-Bucheli et al., 2018). For example, Vm24, which has low picomolar affinity for Kv1.3 (3 pM, Varga et al., 2012) significantly downregulated CD25 and CD40L in T_EM_ lymphocytes at 1 nM concentration (Veytia-Bucheli et al., 2018).

In conclusion, this study reports that the *Pichia* expression system is a powerful method to produce disulfide-rich peptides, and through optimization strategies, overexpression could be enhanced noticeably, making it more cost-effective. rMgTx was produced with a very high yield, as compared with previous reports, by optimizing several factors. The presence of the His-tag on rMgTx was shown to only mildly alter the block equilibrium and binding kinetics for the K^+^ channels. Moreover, both tagged and untagged peptides equipotently reduced the proliferation of CD4^+^ T_EM_ cells. Based on this, tagged rMgTx could be used directly in investigating the role of Kv1.3 and other Kv ion channels in different cells and tissues without removing the tag, thereby reducing the cost and time demand of peptide production.

## Data Availability

The raw data supporting the conclusions of this article will be made available by the authors, without undue reservation.

## References

[B1] Anangi R. Koshy S. Huq R. Beeton C. Chuang W. J. King G. F. (2012). Recombinant Expression of Margatoxin and Agitoxin-2 in Pichia pastoris: an Efficient Method for Production of KV1.3 Channel Blockers. PLoS One 7, e52965. 10.1371/journal.pone.0052965 23300835 PMC3530466

[B2] André N. Cherouati N. Prual C. Steffan T. Zeder-Lutz G. Magnin T. (2006). Enhancing Functional Production of G Protein-Coupled Receptors in Pichia pastoris to Levels Required for Structural Studies via a Single Expression Screen. Protein Sci. 15, 1115–1126. 10.1110/ps.062098206 16597836 PMC2242496

[B3] Bagdány M. Batista C. V. Valdez-Cruz N. A. Somodi S. Rodriguez de la Vega R. C. Licea A. F. (2005). Anuroctoxin, a New Scorpion Toxin of the Alpha-KTx 6 Subfamily, Is Highly Selective for Kv1.3 over IKCa1 Ion Channels of Human T Lymphocytes. Mol. Pharmacol. 67, 1034–1044. 10.1124/mol.104.007187 15615696

[B4] Bartok A. Fehér K. Bodor A. Rákosi K. Tóth G. K. Kövér K. E. (2015). An Engineered Scorpion Toxin Analogue with Improved Kv1.3 Selectivity Displays Reduced Conformational Flexibility. Sci. Rep. 5, 18397. 10.1038/srep18397 26689143 PMC4686915

[B5] Bartok A. Toth A. Somodi S. Szanto T. G. Hajdu P. Panyi G. (2014). Margatoxin Is a Non-selective Inhibitor of Human Kv1.3 K+ Channels. Toxicon 87, 6–16. 10.1016/j.toxicon.2014.05.002 24878374

[B6] Beeton C. Pennington M. W. Norton R. S. (2011a). Analogs of the Sea Anemone Potassium Channel Blocker ShK for the Treatment of Autoimmune Diseases. Inflamm. Allergy Drug Targets 10, 313–321. 10.2174/187152811797200641 21824083 PMC3398462

[B7] Brierley R. A. Davis G. R. Holtz G. C. (1994). Production of Insulin-like Growth Factor-1 in Methylotrophic Yeast Cells. Washington, DC: Google Patents.

[B8] Cahalan M. D. Chandy K. G. (2009). The Functional Network of Ion Channels in T Lymphocytes. Immunol. Rev. 231, 59–87. 10.1111/j.1600-065X.2009.00816.x 19754890 PMC3133616

[B9] Çalık P. Bayraktar E. İnankur B. Soyaslan E. Ş. Şahin M. Taşpınar H. (2010). Influence of pH on Recombinant Human Growth Hormone Production by *Pichia pastoris* . J. Chem. Tech. Biotechnol. 85, 1628–1635.

[B10] Chandy K. G. Wulff H. Beeton C. Pennington M. Gutman G. A. Cahalan M. D. (2004). K+ Channels as Targets for Specific Immunomodulation. Trends Pharmacol. Sci. 25, 280–289. 10.1016/j.tips.2004.03.010 15120495 PMC2749963

[B11] Chang S. C. Huq R. Chhabra S. Beeton C. Pennington M. W. Smith B. J. (2015). N-terminally Extended Analogues of the K^+^ Channel Toxin from Stichodactyla Helianthus as Potent and Selective Blockers of the Voltage-Gated Potassium Channel Kv1.3. FEBS J. 282, 2247–2259. 10.1111/febs.13294 25864722 PMC4472561

[B12] Chi V. Pennington M. W. Norton R. S. Tarcha E. J. Londono L. M. Sims-Fahey B. (2012). Development of a Sea Anemone Toxin as an Immunomodulator for Therapy of Autoimmune Diseases. Toxicon 59, 529–546. 10.1016/j.toxicon.2011.07.016 21867724 PMC3397671

[B13] Clare J. J. Rayment F. B. Ballantine S. P. Sreekrishna K. Romanos M. A. (1991). High-level Expression of Tetanus Toxin Fragment C in *Pichia pastoris* Strains Containing Multiple Tandem Integrations of the Gene. Biotechnology (N Y) 9, 455–460. 10.1038/nbt0591-455 1367310

[B14] Coetzee W. A. Amarillo Y. Chiu J. Chow A. Lau D. Mccormack T. (1999). Molecular Diversity of K+ Channels. Ann. N. Y Acad. Sci. 868, 233–285. 10.1111/j.1749-6632.1999.tb11293.x 10414301

[B15] Cregg J. M. (2007). Introduction: Distinctions between *Pichia pastoris* and Other Expression Systems. Methods Mol. Biol. 389, 1–10. 10.1007/978-1-59745-456-8_1 17951631

[B16] Erdogan A. Schaefer C. A. Schaefer M. Luedders D. W. Stockhausen F. Abdallah Y. (2005). Margatoxin Inhibits VEGF-Induced Hyperpolarization, Proliferation and Nitric Oxide Production of Human Endothelial Cells. J. Vasc. Res. 42, 368–376. 10.1159/000087159 16043967

[B17] Escobar L. Root M. J. Mackinnon R. (1993). Influence of Protein Surface Charge on the Bimolecular Kinetics of a Potassium Channel Peptide Inhibitor. Biochemistry 32, 6982–6987. 10.1021/bi00078a024 7687466

[B18] Escoubas P. Bernard C. Lambeau G. Lazdunski M. Darbon H. (2003). Recombinant Production and Solution Structure of PcTx1, the Specific Peptide Inhibitor of ASIC1a Proton-Gated Cation Channels. Protein Sci. 12, 1332–1343. 10.1110/ps.0307003 12824480 PMC2323924

[B19] Garcia-Calvo M. Leonard R. J. Novick J. Stevens S. P. Schmalhofer W. Kaczorowski G. J. (1993). Purification, Characterization, and Biosynthesis of Margatoxin, a Component of *Centruroides margaritatus* Venom that Selectively Inhibits Voltage-dependent Potassium Channels. J. Biol. Chem. 268, 18866–18874. 10.1016/s0021-9258(17)46707-x 8360176

[B20] Giangiacomo K. M. Ceralde Y. Mullmann T. J. (2004). Molecular Basis of Alpha-KTx Specificity. Toxicon 43, 877–886. 10.1016/j.toxicon.2003.11.029 15208020

[B21] Han S. Hu Y. Zhang R. Yi H. Wei J. Wu Y. (2011). ImKTx88, a Novel Selective Kv1.3 Channel Blocker Derived from the Scorpion *Isometrus maculates* . Toxicon 57, 348–355. 10.1016/j.toxicon.2010.12.015 21194541

[B22] Hofschröer V. Najder K. Rugi M. Bouazzi R. Cozzolino M. Arcangeli A. (2020). Ion Channels Orchestrate Pancreatic Ductal Adenocarcinoma Progression and Therapy. Front. Pharmacol. 11, 586599. 10.3389/fphar.2020.586599 33841132 PMC8025202

[B23] Jensen J. E. Durek T. Alewood P. F. Adams D. J. King G. F. Rash L. D. (2009). Chemical Synthesis and Folding of APETx2, a Potent and Selective Inhibitor of Acid Sensing Ion Channel 3. Toxicon 54, 56–61. 10.1016/j.toxicon.2009.03.014 19306891

[B24] Jiménez-Vargas J. M. Restano-Cassulini R. Possani L. D. (2012). Toxin Modulators and Blockers of hERG K(+) Channels. Toxicon 60, 492–501. 10.1016/j.toxicon.2012.03.024 22497787

[B25] Johnson B. A. Stevens S. P. Williamson J. M. (1994). Determination of the Three-Dimensional Structure of Margatoxin by 1H, 13C, 15N Triple-Resonance Nuclear Magnetic Resonance Spectroscopy. Biochemistry 33, 15061–15070. 10.1021/bi00254a015 7999764

[B26] Kalman K. Pennington M. W. Lanigan M. D. Nguyen A. Rauer H. Mahnir V. (1998). ShK-Dap22, a Potent Kv1.3-specific Immunosuppressive Polypeptide. J. Biol. Chem. 273, 32697–32707. 10.1074/jbc.273.49.32697 9830012

[B27] Kazama I. (2015). Physiological Significance of Delayed Rectifier K(+) Channels (Kv1.3) Expressed in T Lymphocytes and Their Pathological Significance in Chronic Kidney Disease. J. Physiol. Sci. 65, 25–35. 10.1007/s12576-014-0331-x 25096892 PMC10717717

[B28] Khatri N. K. Hoffmann F. (2006). Oxygen-limited Control of Methanol Uptake for Improved Production of a Single-Chain Antibody Fragment with Recombinant *Pichia pastoris* . Appl. Microbiol. Biotechnol. 72, 492–498. 10.1007/s00253-005-0306-1 16532314

[B29] Klint J. K. Senff S. Saez N. J. Seshadri R. Lau H. Y. Bende N. S. (2013). Production of Recombinant Disulfide-Rich Venom Peptides for Structural and Functional Analysis via Expression in the Periplasm of *E. coli* . PloS one 8, e63865. 10.1371/journal.pone.0063865 23667680 PMC3646780

[B30] Kobayashi K. Kuwae S. Ohya T. Ohda T. Ohyama M. Tomomitsu K. (2000). Addition of Oleic Acid Increases Expression of Recombinant Human Serum Albumin by the AOX2 Promoter in *Pichia pastoris* . J. Biosci. Bioeng. 89, 479–484. 10.1016/s1389-1723(00)89100-8 16232781

[B31] Koshy S. Huq R. Tanner M. R. Atik M. A. Porter P. C. Khan F. S. (2014). Blocking KV1.3 Channels Inhibits Th2 Lymphocyte Function and Treats a Rat Model of Asthma. J. Biol. Chem. 289, 12623–12632. 10.1074/jbc.M113.517037 24644290 PMC4007452

[B32] Lam J. Wulff H. (2011). The Lymphocyte Potassium Channels Kv1.3 and KCa3.1 as Targets for Immunosuppression. Drug Dev. Res. 72, 573–584. 10.1002/ddr.20467 22241939 PMC3253536

[B33] Li Y. Zhang B. Chen X. Chen Y. Cao Y. (2010). Improvement of *Aspergillus sulphureus* Endo-Beta-1,4-Xylanase Expression in *Pichia pastoris* by Codon Optimization and Analysis of the Enzymic Characterization. Appl. Biochem. Biotechnol. 160, 1321–1331. 10.1007/s12010-009-8621-0 19412581

[B34] Lobstein J. Emrich C. A. Jeans C. Faulkner M. Riggs P. Berkmen M. (2012). SHuffle, a Novel *Escherichia coli* Protein Expression Strain Capable of Correctly Folding Disulfide Bonded Proteins in its Cytoplasm. Microb. Cel Fact 11, 56. 10.1186/1475-2859-11-56 PMC3526497 22569138

[B35] Macauley-Patrick S. Fazenda M. L. Mcneil B. Harvey L. M. (2005). Heterologous Protein Production Using the *Pichia pastoris* Expression System. Yeast 22, 249–270. 10.1002/yea.1208 15704221

[B36] Mansur M. Cabello C. Hernández L. País J. Varas L. Valdés J. (2005). Multiple Gene Copy Number Enhances Insulin Precursor Secretion in the Yeast *Pichia pastoris* . Biotechnol. Lett. 27, 339–345. 10.1007/s10529-005-1007-7 15834796

[B37] Minjie G. Zhongping S. (2013). Process Control and Optimization for Heterologous Protein Production by Methylotrophic *Pichia pastoris* . Chin. J. Chem. Eng. 21, 216–226.

[B38] Mu X. Kong N. Chen W. Zhang T. Shen M. Yan W. (2008). High-level Expression, Purification, and Characterization of Recombinant Human Basic Fibroblast Growth Factor in *Pichia pastoris* . Protein Expr. Purif. 59, 282–288. 10.1016/j.pep.2008.02.009 18378165

[B39] Nordén K. Agemark M. Danielson J. Å. Alexandersson E. Kjellbom P. Johanson U. (2011). Increasing Gene Dosage Greatly Enhances Recombinant Expression of Aquaporins in *Pichia pastoris* . BMC Biotechnol. 11, 47–12. 10.1186/1472-6750-11-47 21569231 PMC3118338

[B40] Panyi G. Beeton C. Felipe A. (2014). Ion Channels and Anti-cancer Immunity. Philos. Trans. R. Soc. Lond. B Biol. Sci. 369, 20130106. 10.1098/rstb.2013.0106 24493754 PMC3917360

[B41] Panyi G. Possani L. D. Rodríguez de la Vega R. C. Gáspár R. Varga Z. (2006). K+ Channel Blockers: Novel Tools to Inhibit T Cell Activation Leading to Specific Immunosuppression. Curr. Pharm. Des. 12, 2199–2220. 10.2174/138161206777585120 16787250

[B42] Park E. Y. (2006). Enhanced Production of Mouse α-Amylase by Feeding Combined Nitrogen and Carbon Sources in Fed-Batch Culture of Recombinant *Pichia pastoris* . Process Biochem. 41, 390–397.

[B43] Pennington M. W. Chang S. C. Chauhan S. Huq R. Tajhya R. B. Chhabra S. (2015). Development of Highly Selective Kv1.3-blocking Peptides Based on the Sea Anemone Peptide ShK. Mar. Drugs 13, 529–542. 10.3390/md13010529 25603346 PMC4306950

[B44] Péter M. Jr Varga Z. Hajdu P. Gáspár R. Jr Damjanovich S. Horjales E. (2001). Effects of Toxins Pi2 and Pi3 on Human T Lymphocyte Kv1.3 Channels: the Role of Glu7 and Lys24. J. Membr. Biol. 179, 13–25. 10.1007/s002320010033 11155206

[B45] Rashid M. H. Huq R. Tanner M. R. Chhabra S. Khoo K. K. Estrada R. (2014). A Potent and Kv1.3-selective Analogue of the Scorpion Toxin HsTX1 as a Potential Therapeutic for Autoimmune Diseases. Sci. Rep. 4, 4509–9. 10.1038/srep04509 24676092 PMC3968461

[B46] Rodríguez de la Vega R. C. Merino E. Becerril B. Possani L. D. (2003). Novel Interactions between K+ Channels and Scorpion Toxins. Trends Pharmacol. Sci. 24, 222–227. 10.1016/S0165-6147(03)00080-4 12767720

[B47] Rubaiy H. N. (2016). The Therapeutic Agents that Target ATP-Sensitive Potassium Channels. Acta Pharm. 66, 23–34. 10.1515/acph-2016-0006 27029082

[B48] Rudolph R. Lilie H. (1996). *In Vitro* folding of Inclusion Body Proteins. FASEB J. 10, 49–56. 10.1096/fasebj.10.1.8566547 8566547

[B49] Schuh K. Twardzik T. Kneitz B. Heyer J. Schimpl A. Serfling E. (1998). The Interleukin 2 Receptor Alpha chain/CD25 Promoter Is a Target for Nuclear Factor of Activated T Cells. J. Exp. Med. 188, 1369–1373. 10.1084/jem.188.7.1369 9763616 PMC2212486

[B50] Schwartz A. B. Kapur A. Huang Z. Anangi R. Spear J. M. Stagg S. (2020). Olfactory Bulb-Targeted Quantum Dot (QD) Bioconjugate and Kv1. 3 Blocking Peptide Improve Metabolic Health in Obese Male Mice. J. Neurochem. 157, 1876. 10.1111/jnc.15200 32978815 PMC8097972

[B51] Shen B. Cao Z. Li W. Sabatier J. M. Wu Y. (2017). Treating Autoimmune Disorders with Venom-Derived Peptides. Expert Opin. Biol. Ther. 17, 1065–1075. 10.1080/14712598.2017.1346606 28695745

[B52] Sunga A. J. Tolstorukov I. Cregg J. M. (2008). Posttransformational Vector Amplification in the Yeast *Pichia pastoris* . FEMS Yeast Res. 8, 870–876. 10.1111/j.1567-1364.2008.00410.x 18637138

[B53] Tajti G. Szanto T. G. Csoti A. Racz G. Evaristo C. Hajdu P. (2021). Immunomagnetic Separation Is a Suitable Method for Electrophysiology and Ion Channel Pharmacology Studies on T Cells. Channels (Austin) 15, 53–66. 10.1080/19336950.2020.1859753 33356811 PMC7781520

[B54] Tajti G. Wai D. C. C. Panyi G. Norton R. S. (2020). The Voltage-Gated Potassium Channel KV1.3 as a Therapeutic Target for Venom-Derived Peptides. Biochem. Pharmacol. 181, 114146. 10.1016/j.bcp.2020.114146 32653588

[B55] Tarcha E. J. Olsen C. M. Probst P. Peckham D. Muñoz-Elías E. J. Kruger J. G. (2017). Safety and Pharmacodynamics of Dalazatide, a Kv1.3 Channel Inhibitor, in the Treatment of Plaque Psoriasis: A Randomized Phase 1b Trial. PLoS One 12, e0180762. 10.1371/journal.pone.0180762 28723914 PMC5516987

[B56] Tenenholz T. C. Klenk K. C. Matteson D. R. Blaustein M. P. Weber D. J. (2000). “Structural Determinants of Scorpion Toxin Affinity: The Charybdotoxin (α-KTX) Family of K+-channel Blocking Peptides,” in Reviews of Physiology, Biochemistry and Pharmacology, Volume 140 (Springer), 135–185. 10.1007/bfb0035552 10857399

[B57] Teng D. Fan Y. Yang Y. L. Tian Z. G. Luo J. Wang J. H. (2007). Codon Optimization of *Bacillus licheniformis* Beta-1,3-1,4-Glucanase Gene and its Expression in Pichia pastoris. Appl. Microbiol. Biotechnol. 74, 1074–1083. 10.1007/s00253-006-0765-z 17216453

[B58] Toldi G. Bajnok A. Dobi D. Kaposi A. Kovács L. Vásárhelyi B. (2013). The Effects of Kv1.3 and IKCa1 Potassium Channel Inhibition on Calcium Influx of Human Peripheral T Lymphocytes in Rheumatoid Arthritis. Immunobiology 218, 311–316. 10.1016/j.imbio.2012.05.013 22705192

[B59] Toldi G. Legány N. Ocsovszki I. Balog A. (2020). Calcium Influx Kinetics and the Characteristics of Potassium Channels in Peripheral T Lymphocytes in Systemic Sclerosis. Pathobiology 87, 311–316. 10.1159/000509674 32911471

[B60] Tsytsykova A. V. Tsitsikov E. N. Geha R. S. (1996). The CD40L Promoter Contains Nuclear Factor of Activated T Cells-Binding Motifs Which Require AP-1 Binding for Activation of Transcription. J. Biol. Chem. 271, 3763–3770. 10.1074/jbc.271.7.3763 8631992

[B61] Tubert C. Taravini I. R. E. Flores-Barrera E. Sánchez G. M. Prost M. A. Avale M. E. (2016). Decrease of a Current Mediated by Kv1.3 Channels Causes Striatal Cholinergic Interneuron Hyperexcitability in Experimental Parkinsonism. Cell Rep 16, 2749–2762. 10.1016/j.celrep.2016.08.016 27568555

[B62] Varga Z. Gurrola-Briones G. Papp F. Rodríguez de la Vega R. C. Pedraza-Alva G. Tajhya R. B. (2012). Vm24, a Natural Immunosuppressive Peptide, Potently and Selectively Blocks Kv1.3 Potassium Channels of Human T Cells. Mol. Pharmacol. 82, 372–382. 10.1124/mol.112.078006 22622363 PMC3422703

[B63] Vassileva A. Chugh D. A. Swaminathan S. Khanna N. (2001). Expression of Hepatitis B Surface Antigen in the Methylotrophic Yeast *Pichia pastoris* Using the GAP Promoter. J. Biotechnol. 88, 21–35. 10.1016/s0168-1656(01)00254-1 11377762

[B64] Veytia-Bucheli J. I. Jiménez-Vargas J. M. Melchy-Pérez E. I. Sandoval-Hernández M. A. Possani L. D. Rosenstein Y. (2018). Kv1.3 Channel Blockade with the Vm24 Scorpion Toxin Attenuates the CD4+ Effector Memory T Cell Response to TCR Stimulation. Cell Commun Signal 16, 45. 10.1186/s12964-018-0257-7 30107837 PMC6092819

[B65] Wang J.-R. Li Y.-Y. Liu D.-N. Liu J.-S. Li P. Chen L.-Z. (2015). Codon Optimization Significantly Improves the Expression Level of α-amylase Gene from *Bacillus licheniformis* in *Pichia pastoris* . Biomed. Research International 2015, 248680. 10.1155/2015/248680 26171389 PMC4478363

[B66] Wang X. Li G. Guo J. Zhang Z. Zhang S. Zhu Y. (2020). Kv1.3 Channel as a Key Therapeutic Target for Neuroinflammatory Diseases: State of the Art and beyond. Front. Neurosci. 13, 1393. 10.3389/fnins.2019.01393 31992966 PMC6971160

[B67] Waugh D. S. (2011). An Overview of Enzymatic Reagents for the Removal of Affinity Tags. Protein Expr. Purif. 80, 283–293. 10.1016/j.pep.2011.08.005 21871965 PMC3195948

[B68] White C. E. Kempi N. M. Komives E. A. (1994). Expression of Highly Disulfide-Bonded Proteins in *Pichia pastoris* . Structure 2, 1003–1005. 10.1016/s0969-2126(94)00103-0 7881900

[B69] Wulff H. Calabresi P. A. Allie R. Yun S. Pennington M. Beeton C. (2003). The Voltage-Gated Kv1.3 K(+) Channel in Effector Memory T Cells as New Target for MS. J. Clin. Invest. 111, 1703–1713. 10.1172/JCI16921 12782673 PMC156104

[B70] Yadava A. Ockenhouse C. F. (2003). Effect of Codon Optimization on Expression Levels of a Functionally Folded Malaria Vaccine Candidate in Prokaryotic and Eukaryotic Expression Systems. Infect. Immun. 71, 4961–4969. 10.1128/iai.71.9.4961-4969.2003 12933838 PMC187353

[B71] Yang K. C. Nerbonne J. M. (2016). Mechanisms Contributing to Myocardial Potassium Channel Diversity, Regulation and Remodeling. Trends Cardiovasc. Med. 26, 209–218. 10.1016/j.tcm.2015.07.002 26391345 PMC4715991

[B72] Yu M. Wen S. Tan T. (2010). Enhancing Production of *Yarrowia lipolytica* Lipase Lip2 in *Pichia pastoris* . Eng. Life Sci. 10, 458–464. 10.1002/elsc.200900102

[B73] Yu P. Yan Y. Gu Q. Wang X. (2013). Codon Optimisation Improves the Expression of *Trichoderma viride* Sp. Endochitinase in *Pichia pastoris* . Sci. Rep. 3, 3043–3046. 10.1038/srep03043 24154717 PMC3807108

[B74] Zhang T. Gong F. Peng Y. Chi Z. (2009). Optimization for High-Level Expression of the *Pichia guilliermondii* Recombinant Inulinase in *Pichia pastoris* and Characterization of the Recombinant Inulinase. Process Biochem. 44, 1335–1339. 10.1016/j.procbio.2009.07.008

