# Supplementary material for: Optimization of Pichia pastoris Expression System for High-Level Production of Margatoxin
Source: Front Pharmacol. 2021 Sep 29;12:733610. doi: 10.3389/fphar.2021.733610 (PMC8511391; doi:10.3389/fphar.2021.733610)
Supplement: Supplementary file 1 [file DataSheet1.docx]

**Supplementary Data**

**Optimization of *Pichia pastoris* expression system for high-level production of Margatoxin**

**Muhammad Umair Naseem****^1^, Gabor Tajti^1^, Attila Gaspar^2^, Tibor G. Szanto^1^, Jesús Borrego^1^, Gyorgy Panyi^1,*^**

**^1^University of Debrecen, Faculty of Medicine, Department of Biophysics and Cell Biology**

**^2^University of Debrecen, Faculty of Science and Technology, Institute of Chemistry, Department of Inorganic and Analytical Chemistry**


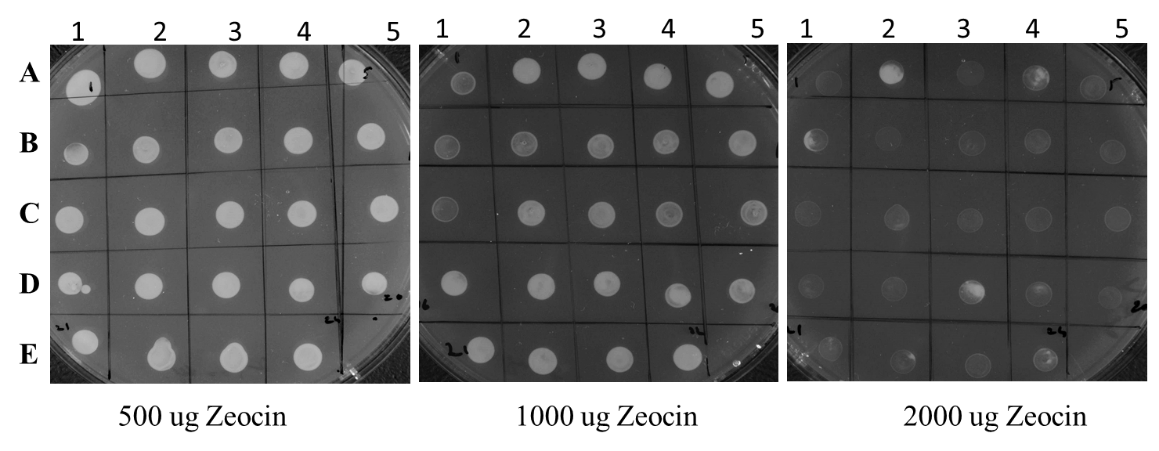


**Suppl. Figure 1:** Growth of *Pichia* X-33 clones on different concentration of Zeocin after 2 days


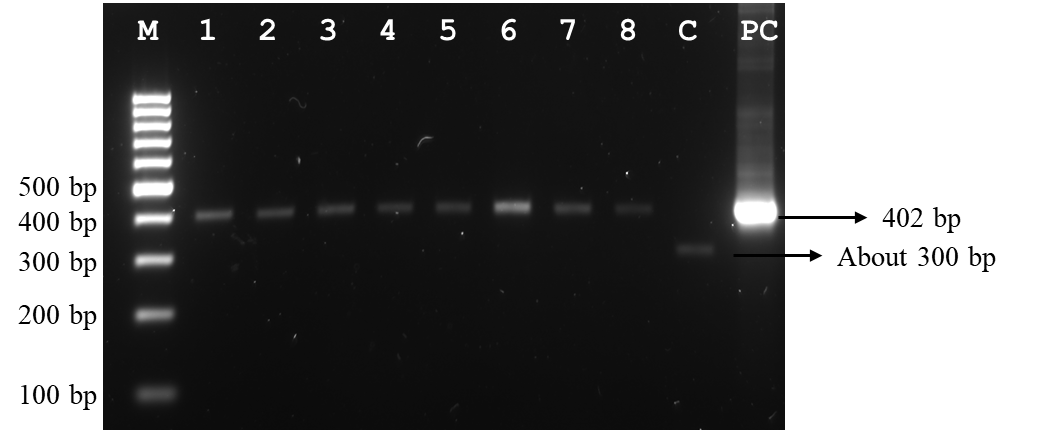


**Suppl. Figure 2:** 2% agarose gel showing the results of colony PCR of *Pichia* X-33 clones transformed with TrMgTx- pPICZαA. M: 100bp DNA marker, **Lane** **1-8**: X-33 clones, **C**: Clone with empty plasmid, **PC**: positive control TrMgTx- pPICZαA plasmid.
